# Design, Synthesis, Antimicrobial Activity and Molecular Docking of New 1,2,4-Triazepine, 1,3,4,6-Oxatriazepine and Pyridazino[1,2-*a*] Pyrimidine Derivatives

**DOI:** 10.3390/ph19010083

**Published:** 2025-12-31

**Authors:** Nasser Amri, Ameen Ali Abu-Hashem

**Affiliations:** Department of Physical Sciences, Chemistry Division, College of Science, Jazan University, P.O. Box 114, Jazan 45142, Saudi Arabia; namri@jazanu.edu.sa

**Keywords:** pyrimidine, pyridazine, pyridazino[1,2-*a*] pyrimidine, 1,2,4-triazepine, 1,3,4,6-oxatriazepine, computational study, molecular docking, antimicrobial activity

## Abstract

**Background**: Recently, compounds such as pyrimidine, pyridazine, 1,2,4-triazepine, 1,3,4,6-oxatriazepine, pyridazino[1,2-*a*]pyrimidine, and pyridazino[1,2-*c*] pyrimidine, as well as their derivatives, have attracted attention due to their diverse biological activities. **Objective**: This study focuses on the synthesis of new heterocyclic compounds that feature a seven-membered ring, including pyridazinopyrimido[2,1-*c*] [1,2,4]triazepine-tetraones **(4)**, pyridazinopyrimidotriazepine-triones **(5–8)**, aminopyri-dazinopyrimido[2,1-*c*][1,2,4]triazepine-tetraone **(9)**, and 6-amino-8-imino-pyridazino pyrimido[2,1-*c*] [1,2,4]triazepine-trione **(10)**. These new compounds were synthesized starting from 1-(4-oxo-1,4-dihydropyrimidine)-1,2-dihydropyridazine-3,6-dione **(3)** and were then evaluated for their antimicrobial activity. **Methods**: A new series of pyridazino[1,2-*a*]pyrimido[2,1-*c*][1,2,4]triazepines and 1,3,4,6-oxatriazepines were synthesized using modern techniques and advanced technology, achieving yields between 72% and 90%. **Results**: All new compounds were confirmed through IR, ^1^H NMR, ^13^C NMR, and mass spectroscopy (MS) and tested for in vitro antimicrobial activity. Compounds **(8-10)** exhibited excellent antimicrobial activity. Computational analysis provided a comprehensive evaluation of the broad-spectrum inhibitory potential of four lead compounds **(6, 8, 9,** and **10)** against key microbial and fungal targets. These compounds demonstrated consistently superior binding affinities compared to control drugs cefotaxime and nystatin across a range of enzymes essential for pathogen viability and virulence. **Conclusions**: The structure–activity relationship (SAR) study established a correlation between the tested compounds and their antimicrobial activity. Molecular docking analysis indicated that the in silico results strongly suggest that compounds **(6, 8, 9,** and **10)** are promising multi-target agents capable of disrupting essential bacterial processes and critical fungal pathways, making them excellent candidates for the development of novel antimicrobial therapeutics. These consistent findings support the conclusion that both practical and theoretical studies of the new compounds align with their antimicrobial effectiveness.

## 1. Introduction

**Figure 1:**
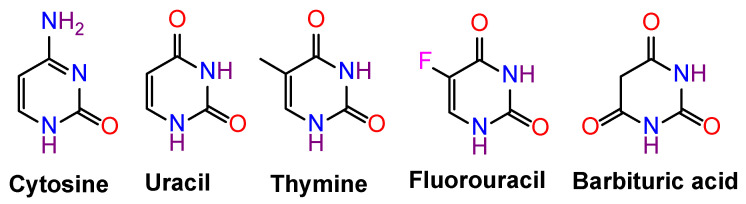
Several pyrimidine derivatives have demonstrated biological activity.

The presence of numerous nitrogen atoms in heterocyclic compounds contributes to their significant biological importance, leading researchers to focus on their synthesis. As a result, nitrogen-containing heterocyclic compounds demonstrate a wide range of biological activities, including the following: In recent decades, researchers have increasingly focused on pyridazine, pyrimidine, and their derivatives due to their notable biological activity. Pyridazine derivatives, in particular, demonstrate a wide range of biological activities [1,2,3], including antimicrobial [4,5], anticancer [6], anti-inflammatory properties [7], and antihypertensive [8]. Pyridazinone and pyridazine derivatives are pharmaceutically acceptable acid-addition salts that serve as active components in cardiovascular tonic formulations, where they enhance cardiac contractility [9,10]. Additionally, thienopyridazines are recognized as anticancer agents [11], while pyridazine derivatives are employed in the treatment of various conditions, including dermatitis, prostate cancer, and dry eye disorders [12]. Pyrimidine is one of nature’s most essential cyclic compounds and is found in various biological systems alongside other compounds, forming a diverse series of cyclic structures. Notable pyrimidine-containing compounds include nucleotides such as uracil, cytosine, and thymine, as well as alloxan. Thiamine, which is structurally related to pyrimidine, represents the chemical structure of vitamin B1 and is used in the treatment of nerve inflammation. Pyrimidine is also present in many pharmaceuticals, often combined with other rings, such as barbiturates. One crucial drug derived from pyrimidine compounds is zidovudine, which is used in the treatment of AIDS, as illustrated in Figure 1.

**Figure 2:**
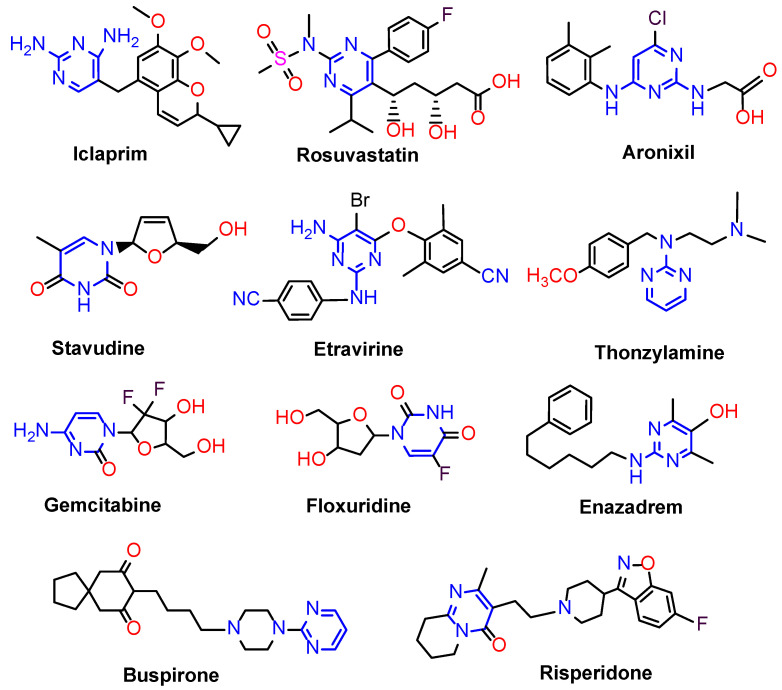
Many drugs generally include a pyrimidine component.

Pyrimidine derivatives and heterocycles have exhibited promising biological activities, including antibacterial [13], antiviral [14], antinociceptive [15], anti-AIDS [16], anti-thrombotic, and antiplatelet effects [17]. Additionally, they serve as inhibitors of multidrug resistance (MDR) [18], findings supported by clinical trials. Pyrimidine-based spirocyclic systems, characterized by a carbon atom shared between two rings, are of significant structural interest [19]. The asymmetric nature of these molecules, due to the presence of a chiral spiro carbon, plays a crucial role in their biological activities. Numerous naturally occurring organic compounds, known as spiro compounds [20], have been studied and shown to have effects. Consequently, spiro compounds are essential, naturally derived substances recognized for their critical biological properties [21,22]. Consequently, the pyrimidine ring plays a crucial role in many medications used to treat various diseases. Some examples of these drugs include Iclaprim (antibiotic), Rosuvastatin (Antilipidemic), Aronixil (antihyperlipidemic), Stavudine (antiretroviral), Etravirine (antiviral), Thonzylamine (antihistaminic), Gemcitabine (anticancer), Floxuridine (anticancer), Enazadrem (anti-psoriatic), Buspirone (anxiolytic), and Risperidone (antipsychotic). These compounds demonstrate the diverse therapeutic potential of pyrimidine derivatives, as shown in Figure 2.

**Figure 3:**
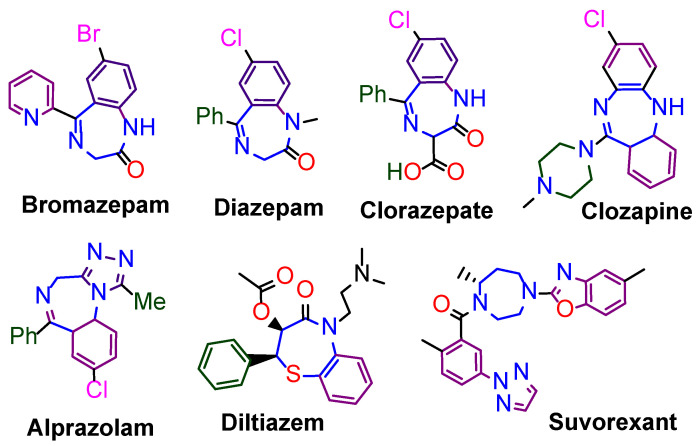
Some medications contain a diazepine structure.

Moreover, seven-membered heterocyclic rings containing nitrogen exhibit various biological activities [23,24,25]. Triazepine derivatives are important seven-membered heterocyclic compounds known for their diverse biological and pharmacological activities. Research over the past decade has highlighted their crucial role in various medications used to treat a range of diseases, particularly those affecting the nervous system. Triazepines and their derivatives exhibit a broad spectrum of biological activities, including antimicrobial, psychotropic, antiviral, anticancer, antisecretory, anti-inflammatory, analgesic, and CCK2 antagonist properties [26,27,28,29,30,31,32,33,34,35,36,37,38,39]. Furthermore, the combination of triazepine and pyrimidine derivatives improves various biological and pharmacological properties, including antifungal, antibacterial, antiviral, antidiabetic, and inhibitory activities [40,41,42]. One type of seven-membered ring that includes nitrogen atoms is the diazepine moiety, which is present in many pharmaceutical compounds [43]. This moiety exhibits a variety of biological activities, including serving as a protein kinase inhibitor [44], a matrix metalloproteinase inhibitor [45], a 5-HT antagonist [46,47], an H3 receptor antagonist [48], a peptidomimetic [49], and an anti-HIV agent [50]. Additionally, some diazepine-containing drugs have been shown to induce DNA strand-breaking activity [51]. These drugs are illustrated in Figure 3.

Over the past decade, our research efforts have led to the publication of numerous scientific articles that benefit both the environment and society. These articles cover a wide range of applications across various fields, particularly in medicine and therapy. They focus on the synthesis of heterocyclic organic compounds and on exploring their biological and pharmacological activities against multiple viruses, microbes, and fungi, among other applications [52,53,54,55,56,57,58,59,60,61,62,63,64,65]. This manuscript explores various safe and environmentally friendly methods for synthesizing new derivatives of 1,2,4-triazepine, 1,3,4,6-oxatriazepine, and pyridazino[1,2-*a*] pyrimidine. These methods are both cost-effective and time-efficient, showing promising applications across multiple fields. Additionally, the antimicrobial activity of these compounds is investigated. This comprehensive study has the potential to contribute to sustainable development by addressing various bacterial and fungal infections, as the newly developed pyridazino[1,2-*a*] pyrimidine derivatives can serve as effective antimicrobial agents. Recent scientific literature has demonstrated that heterocyclic compounds containing pyrimidine, pyrazine, and other ring structures exhibit a wide range of biological activities. One of the most significant findings is their effectiveness against various strains of bacteria and fungi. Computational studies, including molecular docking, have confirmed these results [66,67,68,69,70,71,72,73,74,75,76,77,78,79,80].

## 2. Results and Discussion

### 2.1. Synthesis

**Scheme 1:**
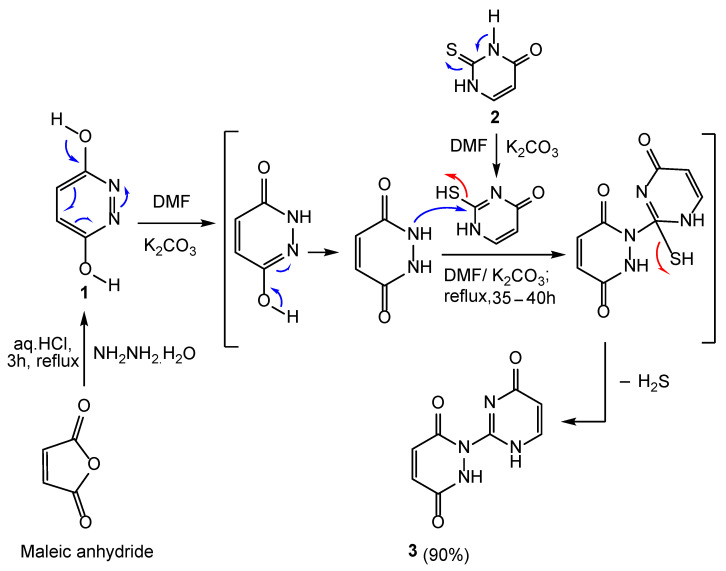
Synthesis of 1-(4-oxo-1,4-dihydropyrimidin-2-yl)-1,2-dihydropyridazine-3,6-dione.

When pyridazine-3,6-diol **(1)** [81] was reacted with 2-thioxo-2,3-dihydropyrimidin-4(1*H*)-one (2-thiouracil, **2**) in dimethylformamide using anhydrous potassium carbonate, it produced 1-(4-oxo-1,4-dihydropyrimidin-2-yl)-1,2-dihydropyridazine-3,6-dione **(3)** through an intermediate compound. The infrared (IR) spectrum of compound **(3)** showed absorption bands at 3340 cm^−1^ and 3325 cm^−1^, indicating the presence of two amine groups (2NH); it also displayed bands at 1688 cm^−1^, 1677 cm^−1^, and 1665 cm^−1^, corresponding to three carbonyl groups. The proton nuclear magnetic resonance (^1^H-NMR) spectrum of compound **(3)** revealed two singlet signals at 9.15 ppm and 10.10 ppm, which correspond to the two protons of the (2NH) groups and are exchangeable with D_2_O; moreover, compound **(3)** exhibited a molecular ion peak at *m*/*z* = 206 (M^+^, 100%). All spectral data, including IR, NMR, MS, and elemental analysis of the new compounds, are detailed in the experimental part (Scheme 1).

**Scheme 2:**
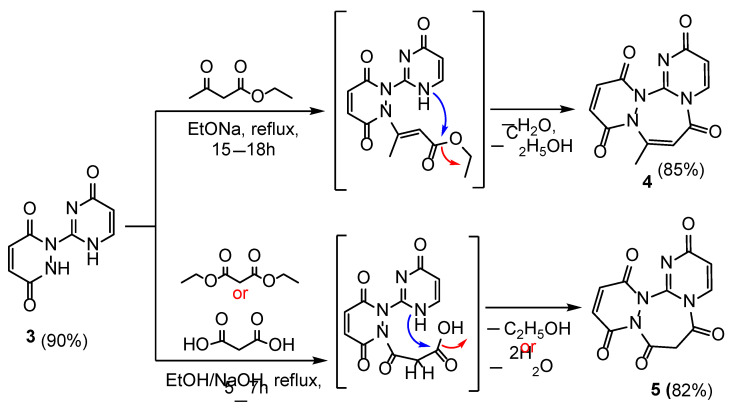
Synthesis of 2*H*,6*H*-pyridazino[1,2-a] pyrimido[2,1-c] [1,2,4] triazepine-tetraone or pentaone derivatives.

The reaction of 1-(4-oxo-1,4-dihydropyrimidin-2-yl)-1,2-dihydropyri-dazine-3,6-dione **(3)** with various α, β-diketones and active methylene reagents such as ethyl acetoacetate, diethyl malonate, malonic acid, acetylacetone, chloro-acetylacetone, propionic anhydride, ethyl cyanoacetate, cyanoacetic acid, and malononitrile was conducted under neat conditions. This resulted in the formation of a range of new heterocyclic products; all achieved with high yields. Furthermore, the condensation of 1-(4-oxo-1,4-dihydropyrimidin-2-yl)-1,2-dihydropyridazine-3,6-dione **(3)** with ethyl-acetoacetate in a sodium ethoxide solution for 15 to 18 h yielded 8-methyl-2*H*,6*H*-pyridazino[1,2-*a*] pyrimido[2,1-*c*] [1,2,4] triazepine-2,6,10,13-tetraone **(4)**. Also, we synthesized the 2*H*,6*H*-pyridazino[1,2-*a*] pyrimido[2,1-*c*] [1,2,4] triazepine-2,6,8,10, 13(7*H*) -pentaone **(5)** via refluxing 1-(pyrimidine) -pyridazine-3,6-dione **(3)** with diethyl malonate or malonic acid in absolute ethanol in the presence of sodium hydroxide for 5 to 7 h. The IR spectrum of compound **(4)** displayed absorption bands at 3100 and 2980 cm^−1^, corresponding to the CH-aryl and CH-alkyl groups, respectively. Additionally, it showed bands at 1690, 1685, 1680, and 1670 cm^−1^, indicating the presence of four carbonyl groups. The ^13^C-NMR spectrum of compound **(4)** revealed absorption signals at *δ* 19.4 and 109.9 ppm, corresponding to two carbon atoms in the (CH_3_) and (CH, triazepine ring) groups. Furthermore, signals at *δ* 152.6, 153.6, 163.4, and 174.7 ppm were attributed to the four carbon atoms of the carbonyl groups. The infrared spectrum of compound **(5)** exhibited absorption bands at *ν* 1700, 1692, 1686, 1681, and 1677, indicating the presence of five carbonyl groups. Additionally, the ^1^H-NMR spectrum of **(5)** revealed distinct signals: a singlet at *δ* 3.72 ppm corresponding to two protons (CH_2_, from the triazepine ring), a doublet at 5.21 ppm and a doublet at 6.80 ppm corresponding to two protons (2CH, from the pyrimidine ring), and another doublet at 7.16 ppm and a doublet at 7.76 ppm corresponding to two protons (2CH, from the pyridazine ring). All spectroscopic data, including IR, NMR, MS, and elemental analysis of the new compounds, are detailed in the experimental section (Scheme 2).

**Scheme 3:**
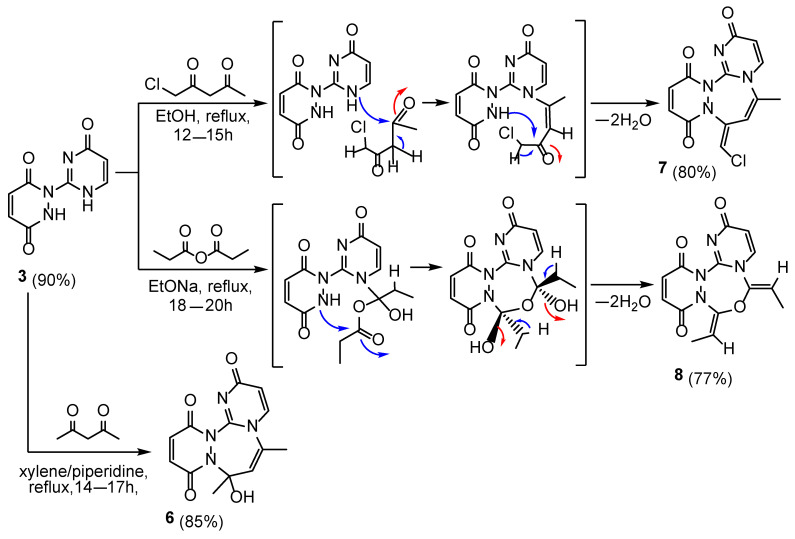
Synthesis of 6,8-diethylidene-pyridazino[1,2-*c*] pyrimido [2,1-*e*] [1,3,4,6] oxatriazepine-2,10,13-trione.

Also, the compound 8-hydroxy-6,8-dimethyl-2*H*,8*H*-pyridazino[1,2-a] pyrimido[2,1-*c*][1,2,4]triazepine-2,10,13-trione **(6)** was synthesized by reacting 1-(4-oxo-1,4-dihydro-pyrimidin-2-yl)-1,2-dihydropyridazine-3,6-dione **(3)** with acetylacetone in dry xylene, using a few drops of piperidine solution. The infrared (IR) spectrum of compound **(6)** showed absorption bands at *ν* = 3510 cm^−1^ for the (OH) group and at ν = 1688, 1684, and 1673 cm^−1^ for the three carbonyl groups. Also, the proton nuclear magnetic resonance (^1^H-NMR) spectrum of compound **(6)** displayed a singlet signal at *δ* 11.52 ppm, corresponding to the one proton of the (OH) group, which is exchangeable with D_2_O. Moreover, treating pyrimidine-1,2-dihydropyridaz-ine-3,6-dione **(3)** with chloroacetylacetone or propionic anhydride in a sodium ethoxide solution, while stirring under reflux for extended periods, produced 8-(chloromethylene)-6-methyl-2*H*,8*H*-pyridazino[1,2-*a*] pyrimido [2,1-c][1,2,4] triazepine-2, 10, 13-trione **(7)** and 6,8-diethylidene-2*H*,6*H*,8*H*-pyridazino [1,2-*c*] pyrimido [2,1-*e*][1,3,4,6] oxatriazepine-2,10,13-trione **(8)**, respectively, by the intermediate outlined in Scheme 3. The infrared (IR) spectrum of compound **(7)** displayed absorption bands at *ν* 1691, 1686, and 1675 cm^−1^, corresponding to the three carbonyl groups. The proton nuclear magnetic resonance (^1^H-NMR) spectrum of **(7)** revealed a singlet signal at *δ* 1.22 ppm for the three protons of the methyl group, and another singlet at *δ* 3.96 ppm for the single proton of the methylene (CH) group in the triazepine ring. In contrast, the IR spectrum of compound **(8)** exhibited absorption bands at *ν* 1696, 1684, and 1671 cm^−1^, also corresponding to its three carbonyl groups. The ^1^H-NMR spectrum of **(8)** displayed a singlet signal at *δ* 3.13 ppm for the six protons from the two methyl groups, as well as two singlets at *δ* 3.81and 4.12 ppm for the two protons of the two methylene (2CH) groups. Mass spectrometry (MS) results for **(7)** and **(8)** showed molecular ion peaks at *m*/*z* 304 (M+, 100%) for (7) and *m*/*z* 300 (M^+^, 100%) for **(8)**. The chemical structures of the new compounds were fully established through IR, NMR, and mass spectrometry, as depicted in Scheme 3. 

**Scheme 4:**
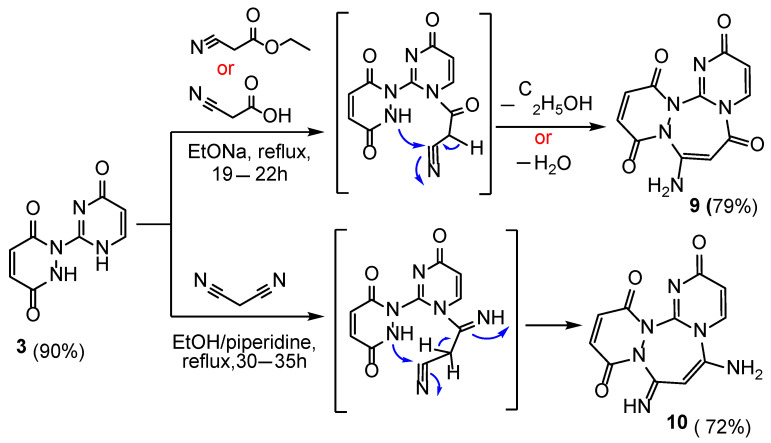
Synthesis of 6-amino-8-imino-pyridazino[1,2-*a*] pyrimido [2,1-c] [1,2,4] triazepine-trione derivatives.

In addition, when 1-(4-oxo-1,4-dihydropyrimidin-2-yl)-1,2-dihydropyri-dazine-3,6-dione **(3)** was treated with ethyl cyanoacetate or cyanoacetic acid in sodium ethoxide in ethanol, it yielded 8-amino-2*H*,6*H*-pyridazino[1,2-a]pyrimido[2,1-c][1,2,4]triazepine-2,6, 10,13-tetraone **(9)** via an intermediate. In addition, the reaction of compound **(3)** with malononitrile in absolute ethanol, using piperidine as a catalyst, resulted in the formation of 6-amino-8-imino-2*H*,8*H*-pyridazino [1,2-*a*] pyrimido[2,1-*c*] [1,2,4] triazepine-2, 10, 13-trione **(10)** in good yield, also through an intermediate in a stepwise process. The compounds **(9)** and **(10)** were analyzed using infrared (IR) and proton (^1^H) and carbon (^13^C) nuclear magnetic resonance (NMR) spectroscopy. The IR spectrum of compound **(9)** displayed absorption bands at *ν* 3425 cm^−1^ for the amino (NH_2_) group and additional bands at 1698, 1690, 1684, and 1677 cm^−1^ corresponding to four imidic carbonyl groups. The ^1^H-NMR spectrum of **(9)** showed a singlet signal at *δ* 7.25 ppm for the two protons from the amino group, which is exchangeable with deuterated water (D_2_O). The IR spectrum of compound **(10)** exhibited a broad band at *ν* 3430 and 3350 cm^−1^, attributed to the amino (NH_2_) and amine (NH) groups, respectively. Additionally, it displayed sharp peaks at *ν* 1700, 1695, and 1682 cm^−1^, corresponding to three imidic carbonyl groups. The ^1^H-NMR spectrum of **(10)** revealed two singlet signals at *δ* 6.10 and 9.22 ppm, which correspond to the protons in the NH_2_ and NH groups, respectively. Evidence of D_2_O exchange confirmed the presence of these groups. Mass spectrometry identified the molecular ion peaks for compound **(10)** at *m*/*z* = 272 (M^+^, 100%). The experimental section presents the results of the spectroscopy analysis for these new compounds, as illustrated in Scheme 4.

### 2.2. Biological Activities

#### 2.2.1. Biological Screening

Newly synthesized pyridazino[1,2-*a*]pyrimido[2,1-c][1,2,4]triazepine and pyridazi- no [1,2-*c*] pyrimido[2,1-*e*] [1,3,4,6] oxatriazepine derivatives were tested in vitro for their antimicrobial activity at the lowest inhibitory concentration against various types of bacteria and fungi [5,53,55,56,59,81]. The bacteria tested included Gram-negative species like *Klebsiella pneumoniae* and *Escherichia coli*, as well as Gram-positive species such as *Streptococcus pyogenes* and *Staphylococcus aureus*. The fungi tested included *Candida albicans*, *Curvularia lunata*, *Alternaria alternate*, and *Aspergillus niger*. Several compounds, specifically compounds **10**, **9,** and **8**, demonstrated the most potent antibacterial and antifungal activity, comparable to the standard drugs cefotaxime sodium (minimum inhibitory concentration [MIC] = 1–2 μmol mL^−1^) and nystatin (MIC = 1–3 μmol mL^−1^), respectively. Additionally, compounds **6**, **7**, and **5** exhibited moderate antimicrobial activity, while the remaining compounds showed weak antimicrobial activity. The complete results are presented in Table 1 and Table 2.

#### 2.2.2. Structural Activity Relationship (SAR)

The biological activity of the new compounds as antimicrobials against various bacteria and fungi was examined. The results revealed significant variations in the activity of some compounds, which can be attributed to several factors discussed below. Some compounds show vigorous activity and effectiveness against various types of bacteria and fungi like 6-amino-8-imino-2*H*,8*H*-pyridazino[1,2-*a*]pyrimido[2,1-*c*][1,2,4]triazepine-2,10,13-tri- one **(10)**, 8-amino-2*H*,6*H*-pyridazi-no[1,2-*a*]pyrimido[2,1-*c*][1,2,4] triazepine- 2,6,10, 13-tetraone **(9)**, 6,8-diethylidene-2*H*,6*H*,8*H*-pyridazino[1,2-*c*]pyrimido [2,1-*e*][1,3,4,6] oxatriazepine-2,10,13-trione **(8),** and 8-hydroxy-6,8-dimethyl -2*H*,8*H*-pyridazino[1,2-*a*] pyrimido[2,1-*c*] [1,2,4] triazepine-2,10,13-trione **(6)**. Also, some products show moderate antibacterial and antifungal activity compared to standard drugs such as Cefotaxime sodium and Nystatin, like 8-(chloromethylene)-6-methyl-2*H*,8*H*-pyridazino[1,2-*a*]pyrimido[2,1-*c*][1,2,4]triazepine-2, 10, 13-trione **(7)**, 2*H*,6*H*-pyridazino[1,2-*a*] pyrimido[2,1-*c*] [1,2, 4] triazepine-2,6,8,10,13(7*H*)-pentaone **(5)** and 8-methyl-2*H*,6*H*-pyridazino [1,2-*a*] pyrimido [2,1-*c*] [1,2,4] triazepine-2,6,10,13-tetraone **(4)**. We examined the connection between the practical outcomes of these compounds and their chemical structures, emphasizing prior scientific research. Our findings are as follows. The compounds demonstrating the best antimicrobial activity contained functional groups related to pyridazino[1,2-*a*] pyrimidine or pyri- dazino[1,2-*c*] pyrimidine, including 1,2,4-triazepine, 1,3,4,6-oxatriazepine, amine, imine, ethylidene, hydroxy, and methyl groups. Additionally, they included heteroatoms such as nitrogen and oxygen. Previous studies have demonstrated that the presence of similar heterocycles and functional groups in various compounds can influence the types of bacteria and fungi present. The results of microbial testing on the activity of newly prepared compounds against multiple strains of bacteria and fungi in vitro indicate that all these species were effectively killed at the minimum concentration of the active compounds, specifically compounds 6, 8, 9, and 10, after a short period. These compounds demonstrated the highest efficacy against the selected bacterial and fungal strains. Furthermore, a comparison of the results from these compounds with those of standard drugs yielded similar outcomes. These experimental findings are also consistent with the theoretical computational studies conducted in this research, suggesting that the pyrimidine, pyridazine, triazepine, and oxatriazepine rings play a significant role in the observed results. Previous scientific studies support this conclusion [5,53,55,56,59,66,67,68,69,70,71,72,73,74,75,76,77,78,79,80,81,82,83,84,85,86].

**Figure 4:**
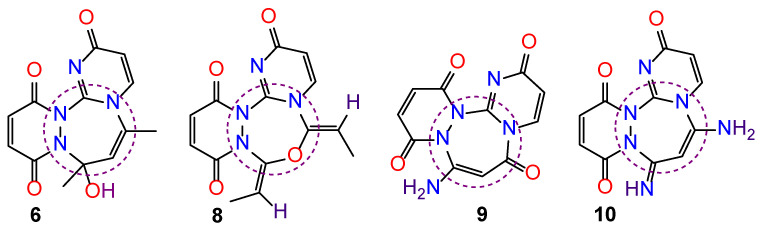
Pyridazinopyrimidine-1,2,4-triazepines and 1,3,4,6-oxatriazepines exhibit potent antimicrobial activity.

This supports the validity of the results obtained for the new compounds developed in this study as antimicrobial agents, as shown in Figure 4.

### 2.3. Computational Studies (Molecular Modeling)

Molecular docking is a widely used technique for predicting how small-molecule therapeutic compounds interact with their protein targets. This method provides valuable insights into the compounds’ affinity and activity, making it essential for rational drug design. Given the biological and pharmacological importance of docking studies, significant efforts have been made to improve predictive algorithms [87]. Docking enables the assessment of potential interactions between synthesized compounds and protein receptors, clarifying their binding behaviors and possible antibacterial effects [52,53,54,56,62,88]. This study observed high-affinity binding to several bacterial targets, including Gyrase B in (*Bacillus pumilus*) and (*E. coli*), Sortase A in (*Streptococcus pyogenes*), and the KPC-2 carbapenemase in (*Klebsiella pneumoniae*). Furthermore, significant inhibition was predicted against several fungal enzymes, such as the Fdc1 enzyme in *Aspergillus niger*, the sterol 14α-demethylase (CYP51) in *Candida albicans*, and the Fusicoccin-2,10 (14)-diene synthase virulence factor in *Alternaria alternata.*

#### 2.3.1. Analysis of Computational Results

##### Docking and Molecular Interaction with Gyrase B of *S. aureus* (PDB: ID 4URM)

**Figure 5:**
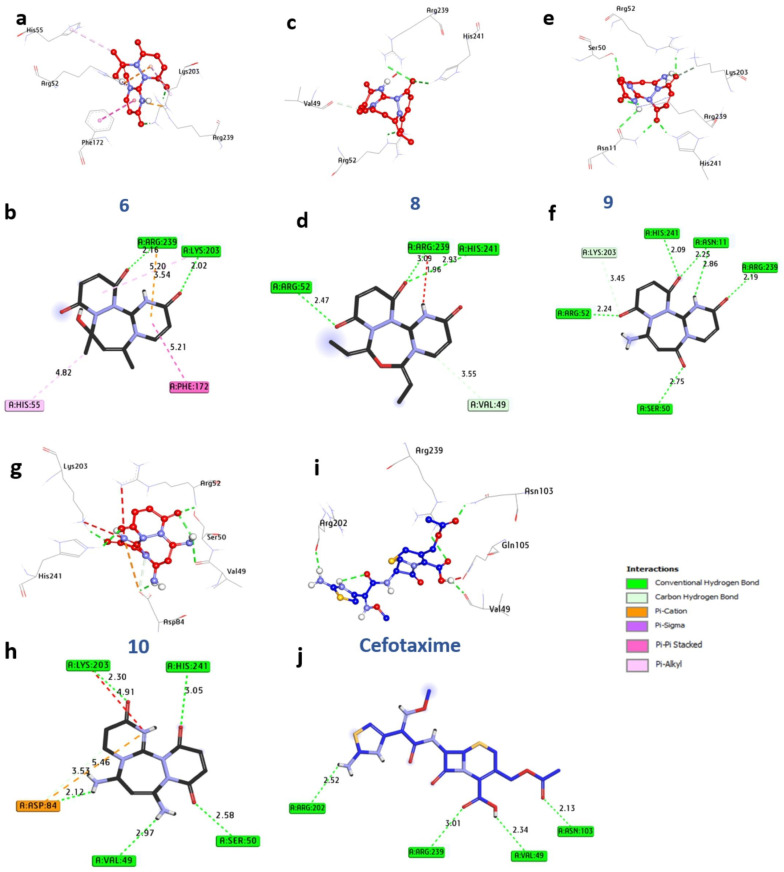
Three-dimensional representations of compound conformations at the binding pocket of Gyrase B of *Staphylococcus aureus* (PDB: ID 4URM): (**a**,**b**) **6**, (**c**,**d**) **8**, (**e**,**f**) **9**, (**g**,**h**) **10**, (**i**,**j**) Cefotaxime.

**Graph 6. 8**, **9**, and **10** as particularly promising, with binding energies of −9.30, −9.30, −9.20, and −9.10 kcal/mol, respectively. These values exceed the reference affinity of Cefotaxime, which is −7.10 kcal/mol. A critical hydrogen bond was observed between compound 6 and the catalytic residue Lys203, while compounds **8**, **9**, and **10** formed similar bonds with residues such as Arg239, Arg52, His241, Asn11, Ser50, Val49, and Asp84. Additionally, further stabilization came from various interactions, including hydrophobic, carbon–hydrogen, Pi–Pi stacking, and Pi–cation interactions with key residues like His55, Phe172, and Arg239 (Table 3 and Figure 5). These interactions, particularly with the catalytic residues Lys203 and Arg239, significantly enhance ligand binding. Overall, the findings highlight the strong inhibitory potential of these compounds against Gyrase B in *B. pumilus*. This research aligns with recent studies that have applied molecular docking to investigate antibacterial mechanisms, focusing on compound–protein interactions and examining small-molecule inhibition of Gyrase B in *B. subtilis* [52,53,54,56,62].

##### Docking and Molecular Interaction Studies with DNA Gyrase of *E. coli*

**Figure 6:**
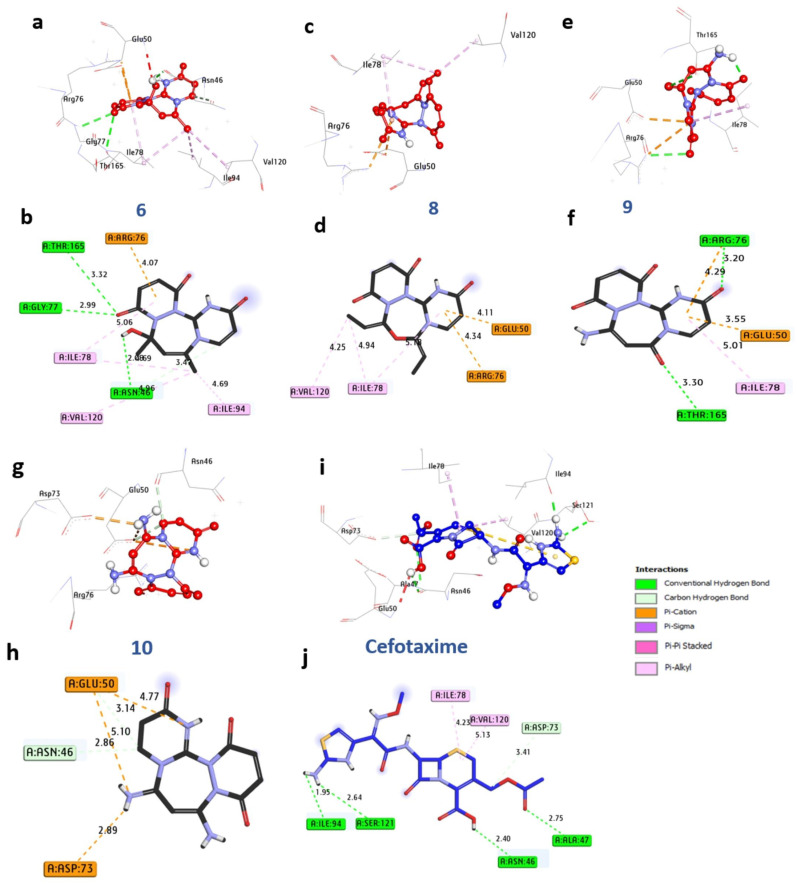
Three-dimensional representations of compounds conformations at the binding pocket of GyrB24 of *E. coli* (PDB: ID 7P2M): (**a**,**b**) **6**, (**c**,**d**) **8**, (**e**,**f**) **9**, (**g**,**h**) **10**, (**i**,**j**) Cefotaxime.

DNA gyrase is an essential enzyme in bacteria, particularly in *E. coli*, as it plays a crucial role in sustaining DNA replication and transcription by resolving topological strain. Molecular docking analysis has identified compounds **6**, **8**, **9**, and **10** as the most potent inhibitors, with binding affinities of −7.60, −7.40, −7.80, and −7.30 kcal/mol, respectively. Each of these affinities surpasses that of the control drug, Cefotaxime, which has a binding affinity of −6.20 kcal/mol. Compounds **6** and **9** form key hydrogen bonds with critical catalytic residues, including Gly77, Arg76, Asn46, and Thr165. These interactions are crucial for stabilizing the ligands within the enzyme’s active site. Additionally, the binding is reinforced by various hydrophobic interactions—such as alkyl bonds with Val120, Ile78, and Ile94; carbon–hydrogen bonds with Asn46; and Pi–cation bonds with residues like Arg76 and Asp73 (see Table 4 and Figure 6). The significant involvement of catalytic residues Gly77, Thr165, and Gly117 underlines the enhanced binding affinity of these compounds. Collectively, these findings indicate that compounds **6**, **8**, **9**, and **10** are promising candidates for inhibiting *E. coli* DNA gyrase. This conclusion is further supported by recent research [88], which integrated antimicrobial assays with in silico docking to validate DNA gyrase as a viable target for small-molecule inhibition.

##### Docking and Interaction of *Streptococcus pyogenes* Sortase A (spySrt A)

**Figure 7:**
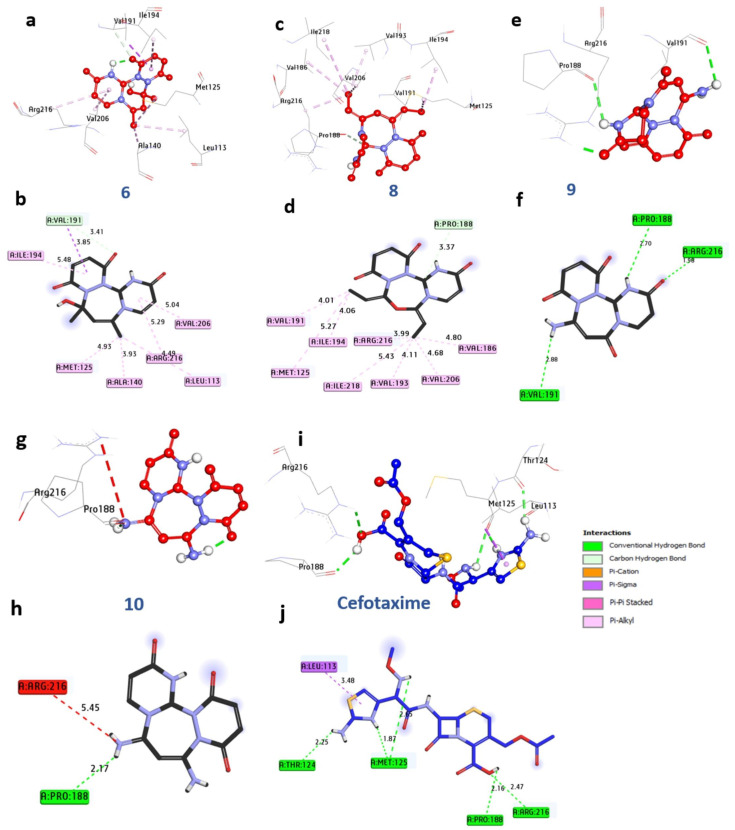
Three-dimensional representations of compounds at the binding pocket of *Streptococcus pyogenes* Sortase A (spySrtA) (PDB: ID 8T8G): (**a**,**b**) **6**, (**c**,**d**) **8**, (**e**,**f**) **9,** (**g**,**h**) **10,** (**i**,**j**) Cefotaxime.

Sortase A from *Streptococcus pyogenes* is a critical transpeptidase that anchors virulence-related proteins to the bacterial cell wall. Due to its essential role in this bacterium’s pathogenicity, Sortase A is a prime target for anti-virulence therapies. Molecular docking studies have identified compounds **6**, **8**, **9**, and **10** as potent inhibitors, with binding affinities of −6.40, −6.80, −6.10, and −6.00 kcal/mol, respectively. Notably, compound 8 shows a stronger binding affinity than the control drug Cefotaxime (−6.10 kcal/mol). Significant stabilizing interactions involve hydrogen bonds between compounds **9** and **10** and the catalytic residues Val191, Pro188, and Arg216. These interactions are further reinforced by a network of hydrophobic contacts, including alkyl bonds with residues Val206, Arg216, and Ile194, as well as Pi–sigma and C-H bonds with Val191 and Pro188 (Table 5 and Figure 7). The key catalytic residues Pro188, Ile194, Met125, and Val191 are crucial for enhancing ligand binding, highlighting their functional significance. Supported by complementary in vitro antibacterial data, these computational insights suggest that these compounds are promising candidates for inhibiting this vital virulence factor. This research corroborates the findings [52,53,54], which validated similar compounds against *S. pyogenes* Sortase A, demonstrating the effective partnership between computational predictions and experimental validation in the development of novel anti-virulence agents.

##### Docking and Interaction with the KPC-2 Carbapenemase of *K. pneumoniae*

**Figure 8:**
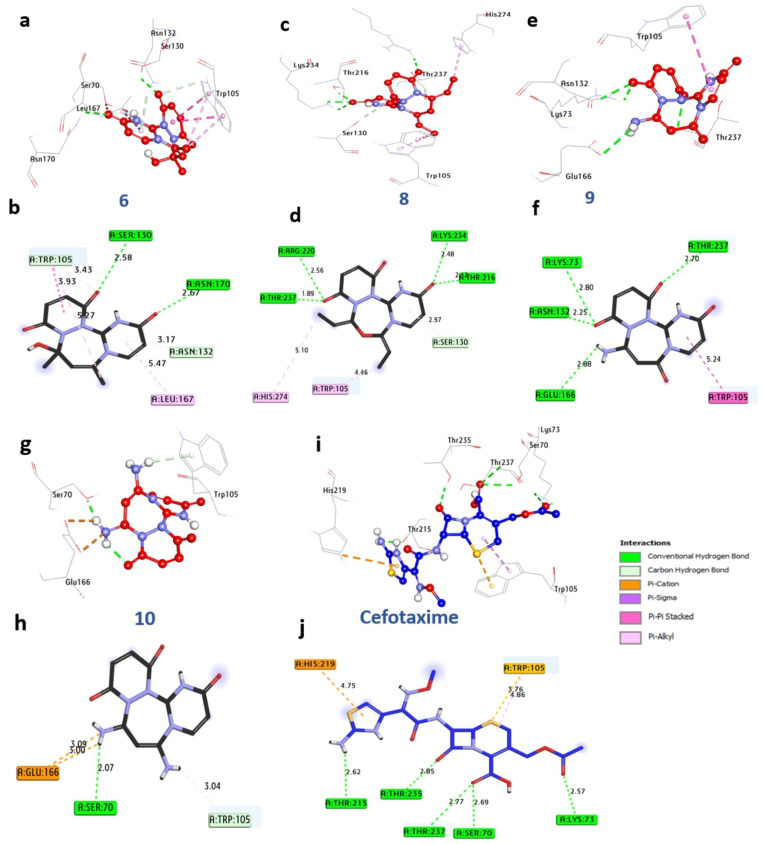
Three-dimensional representations of compound conformations at the binding pocket of KPC-2 carbapenemase of *K. pneumoniae* (PDB: ID 2OV5): (**a**,**b**) **6**, (**c**,**d**) **8**, (**e**,**f**) **9,** (**g**,**h**) **10,** (**i**,**j**) Cefotaxime.

KPC-2 (*Klebsiella pneumoniae* carbapenemase-2), a Class A serine-β-lactamase, poses a serious threat to modern healthcare by inactivating last-resort antibiotics. A computational docking analysis shows that compounds **6**, **8**, **9**, and **10** are effective inhibitors, with binding energies of −8.10, −7.90, −8.90, and −7.30 kcal/mol, respectively. All these energies surpass that of the control drug, Cefotaxime, which has a binding energy of −7.40 kcal/mol. These compounds interact with key catalytic residues, such as Ser130, Glu166, and Ser70, which are vital for substrate coordination. Their binding is further strengthened by a complex network of hydrophobic interactions, including alkyl bonds with Trp105 and His274, Pi–Pi stacking interactions with Trp105, Pi–cation bonds with Glu166, and carbon–hydrogen bonds with Asn132. Notably, the residues Thr237, Ser70, and Glu166 at the catalytic site play an essential role in increasing ligand affinity (Table 6 and Figure 8). Overall, these detailed molecular interactions suggest that the antibacterial activity of these compounds results from direct inhibition of the KPC-2 carbapenemase in *K. pneumoniae*. This aligns with previous findings [52,53,54,56,62], which also used molecular docking to identify and confirm small-molecule inhibitors targeting this crucial enzyme.

##### Docking and Interaction with *A. niger* Fdc1 (4ZA5)

**Figure 9:**
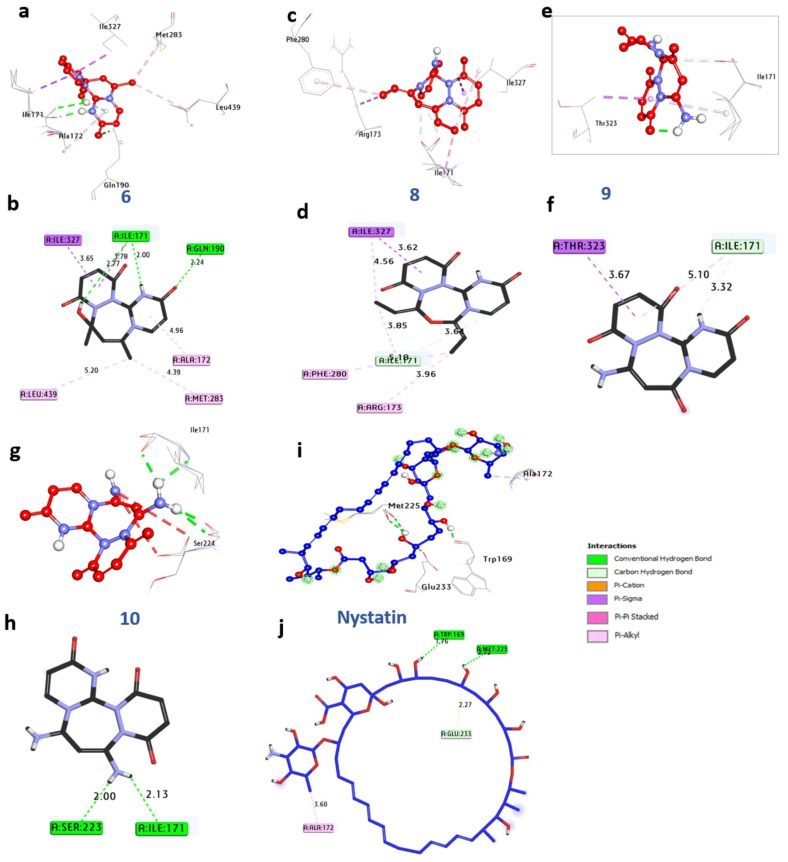
Three-dimensional representations of compounds conformations at the binding pocket of *A. niger* Fdc1 (PDB: ID 4ZA5): (**a**,**b**) **6**, (**c**,**d**) **8**, (**e**,**f**) **9**, (**g**,**h**) **10**, (**i**,**j**) Nystatin.

Fdc1 is a fungal decarboxylase capable of biosynthesizing styrene from renewable cinnamic acid, providing a sustainable alternative for plastic production. Molecular docking analysis identified compounds **6**, **8**, **9**, and **10** as potential inhibitors, with binding affinities of −12.40, −12.00, −9.00, and −9.60 kcal/mol, respectively. These values surpass the binding affinity of the reference compound Nystatin, which is −8.10 kcal/mol. Significant hydrogen bonds are formed by compounds 6 and 10 with residues in the catalytic pocket, including Gly138, Val134, and Gln133. Additionally, various hydrophobic interactions further stabilize the binding; these include alkyl bonds with residues such as Leu77, Ala165, and Tyr137, a pi–cation bond with Tyr168, and carbon–hydrogen bonds with Tyr160 and Glu132. The key residues Gly138, Gln133, and Ala165 within the active site notably enhance ligand affinity, implying that these compounds inhibit Fdc1 by disrupting its substrate recognition or catalytic mechanism (Table 7 and Figure 9). Collectively, these molecular interactions suggest that the compounds exert their antimicrobial effects by targeting and blocking *A. niger* Fdc1. This computational evidence aligns with previous studies that identified potent small-molecule interactors with Fdc1 [52,53,54,56,62,88], which employed docking to design novel inhibitors. These findings reinforce the enzyme’s potential as a therapeutic target.

##### Docking and Interaction with Sterol 14-alpha Demethylase (CYP51) of *C. albicans*

**Figure 10:**
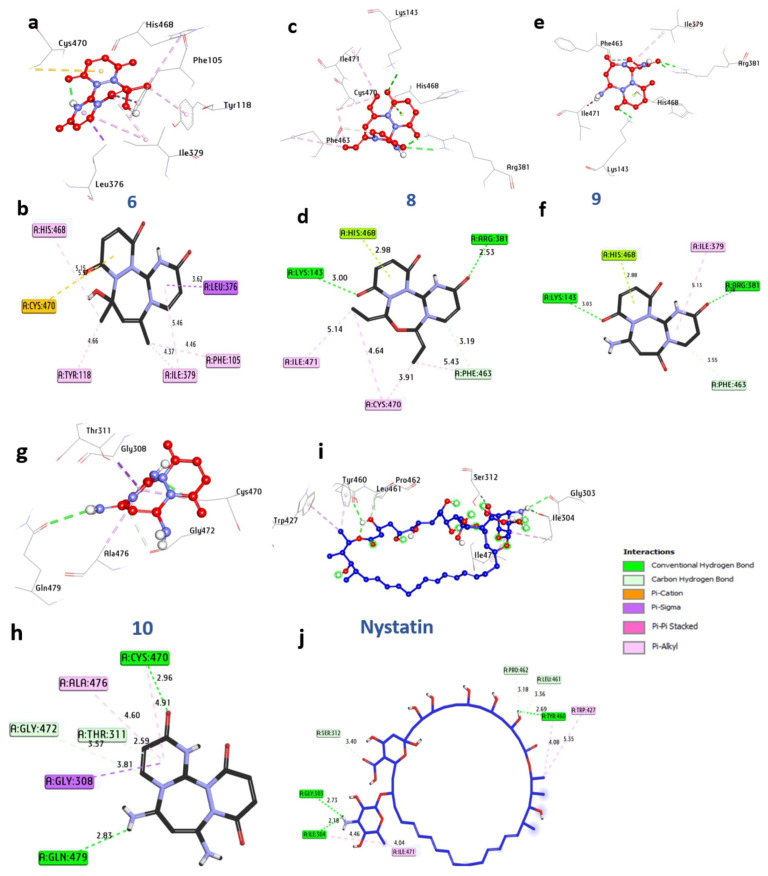
Three-dimensional representations of compounds at the binding pocket of Sterol 14-alpha demethylase of *C. albicans* (PDB: ID 5TZ1): (**a**,**b**) **6**, (**c**,**d**) **8**, (**e**,**f**) **9**, (**g**,**h**) **10**, (**i**,**j**) Nystatin.

Sterol 14-alpha demethylase (CYP51) is an essential enzyme in the biosynthesis of ergosterol in fungi, a process critical for maintaining cell membrane integrity. This enzyme removes the 14α-methyl group from lanosterol, making it a key target for antifungal treatments. Molecular docking analyses have identified compounds **6**, **8**, **9**, and **10** as potent inhibitors of CYP51, with respective binding affinities of −7.50, −7.60, −6.90, and −6.90 kcal/mol, all significantly higher than that of the reference drug, Nystatin, which has a binding affinity of −6.20 kcal/mol. Notably, compounds 6 and 8 form stabilizing hydrogen bonds with critical catalytic residues such as Pro462, Gly308, Thr311, and Leu376. Furthermore, a network of hydrophobic interactions stabilizes the ligand–enzyme complexes. These interactions include alkyl bonds with residues like Ile379, Leu376, and Phe463, a sulfur bond with Cys470, a pi–sigma interaction with Thr311, and carbon–hydrogen bonds with Pro375 and Gly308. The catalytic residues Pro462, Leu376, Thr311, and Gly308 are central to the increased binding affinity observed with these compounds. These computational insights identify these compounds as promising candidates for inhibiting CYP51 in C. albicans, as detailed in Table 8 and Figure 10. The findings are consistent with previous research [89], which effectively employed molecular docking to identify and validate inhibitors for other microbial enzyme targets, thus underscoring the broader applicability of this method in antimicrobial drug development.

##### Docking and Interaction with AaTPS of *Alternaria alternata*

**Figure 11:**
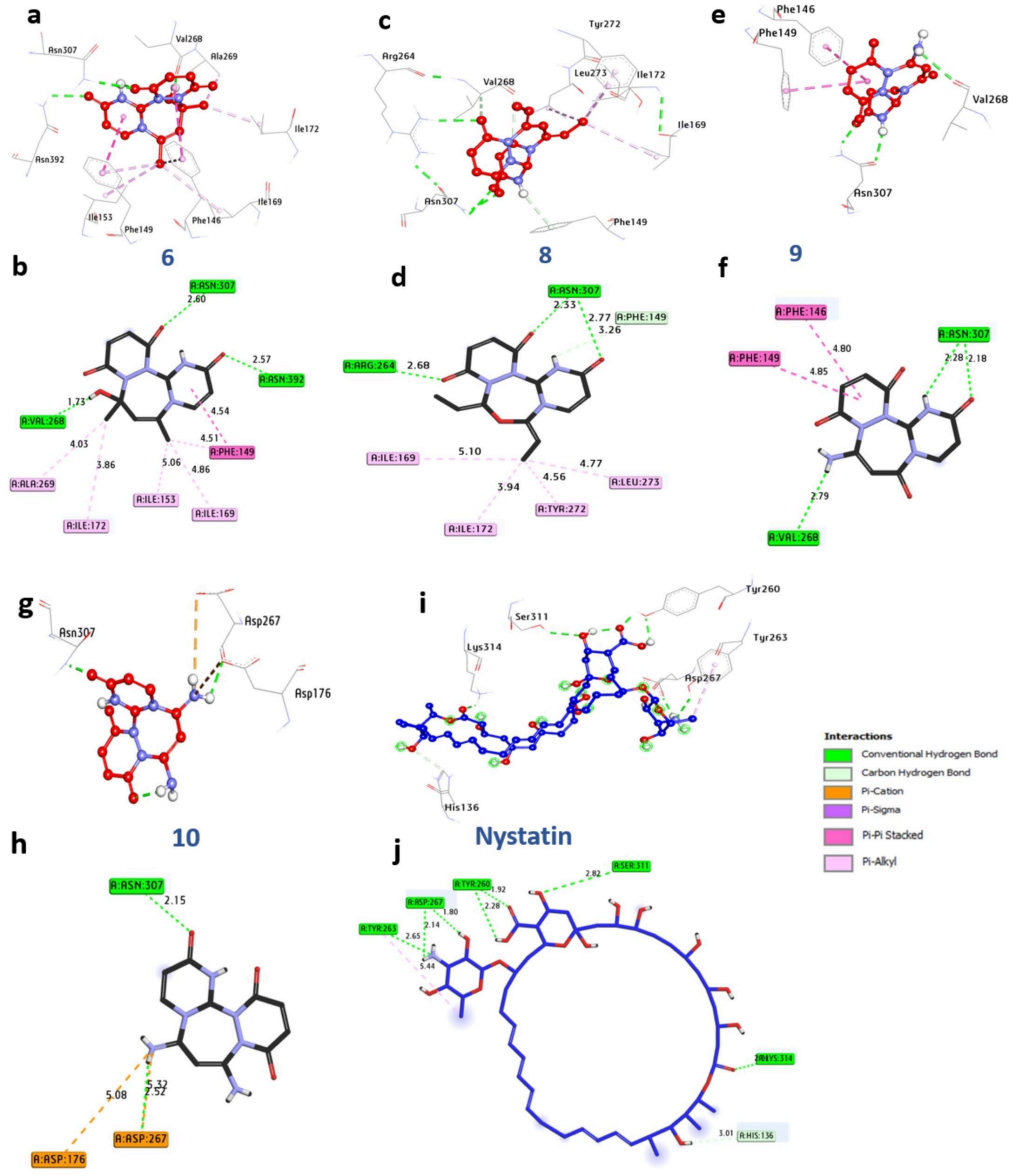
Three-dimensional representations of compounds at the binding pocket of AaTPS of *Alternaria alternata* (PDB: ID 6LCD): (**a**,**b**) **6**, (**c**,**d**) **8**, (**e**,**f**) **9**, (**g**,**h**) **10**, (**i**,**j**) Nystatin.

The Fusicocca-2,10(14)-diene synthase from *Alternaria alternata* is a key enzyme in fungal terpene biosynthesis and a significant virulence factor. This enzyme permanently activates host plasma membrane H+-ATPases, disrupts stomatal function, and triggers plant cell death, thereby aiding fungal invasion. Molecular docking analysis has identified compounds **6**, **8**, **9**, and **10** as potent inhibitors of this synthase, with binding affinities of −8.40, −8.50, −8.20, and −8.30 kcal/mol, respectively. Each of these compounds surpasses the reference compound Nystatin, which has a binding affinity of −7.50 kcal/mol. The identified compounds form vital hydrogen bonds with key catalytic residues, including Asn307, Val268, and Asp267. Binding is further stabilized by a network of hydrophobic interactions, which include alkyl bonds with residues such as Ile172 and Tyr272, Pi–Pi stacking interactions with Phe146 and Phe149, Pi–cation bonds with Asp267 and Asp176, and carbon–hydrogen bonds with Phe149. The catalytic site residues Asn307, Val268, Phe149, and Ile172 play a central role in enhancing ligand affinity (Table 9 and Figure 11). These interactions emphasize the potential of these compounds to inhibit a critical virulence factor in A. alternata. This research aligns with the methodologies [89], who successfully applied molecular docking to identify inhibitors for other microbial targets, reaffirming the efficacy of this strategy in developing antifungal agents.

## 3. Experimental Section

In collaboration with our research team, we developed a research plan to synthesize new heterocyclic compounds, including 1,2,4-triazepine, 1,3,4,6-oxatriazepine, and derivatives of pyridazino[1,2-*a*] pyrimidine. We will also investigate the antimicrobial biological activity of these new compounds in the laboratory. Through consistent effort, we successfully implemented the plan we developed.

### 3.1. General Information

All melting points were determined using an Electrothermal IA 9100 series digital melting point apparatus (Shimadzu, Tokyo, Japan). Elemental analyses were performed using a Vario EL (Elementar, Langenselbold, Germany). The microanalytical data were processed at the Microanalytical Centre of the Faculty of Science at Cairo University and the National Research Centre. The IR spectra (KBr disk) were recorded using a Perkin–Elmer 1650 spectrometer (Waltham, MA, USA). NMR spectra were obtained using a JEOL 270 MHz and a JEOL JMS-AX 500 MHz (JEOL, Tokyo, Japan) spectrometer with Me4Si as the internal standard. Mass spectra were recorded on an EI-MS-QP 1000 EX instrument (Shimadzu, Tokyo, Japan) at 70 eV. Biological evaluations of new antimicrobial compounds were conducted in the Antimicrobial Unit of the Department of Chemistry for Natural and Microbial Products at the National Research Centre in Egypt. All starting materials, solvents, and reagents were sourced from Sigma-Aldrich (St. Louis, MO, USA).

### 3.2. Synthesis of 1-(4-oxo-1,4-Dihydropyrimidin-2-yl)-1,2-dihydropyridazine-3,6-dione (**3**)

A mixture of pyridazine-3,6-diol (**1**) (1.12 g, 10 mmol) and 2-thioxo-2,3-dihydropyrimidin-4(1*H*)-one (2-thiouracil, **2**) (1.28 g, 10 mmol) was refluxed in dimethylformamide (60 mL) with anhydrous potassium carbonate (1.38 g, 10 mmol) for 35–40 h, monitored by thin-layer chromatography (TLC). After the reaction, the mixture was cooled, and the precipitate formed was filtered. This precipitate was washed with cooled water and ethanol, then dried and crystallized from dioxane, resulting in the production yellow crystals of compound **(3)** in good yield (90%) and a melting point >350 °C (melted); IR (*ν*, cm^−1^) KBr: 3340, 3325 (2NH), 3095 (CH aryl), 1688, 1677, 1665 (3CO, three imidic carbonyl group), 1635(C=N), 1590 (C=C); ^1^H NMR (DMSO-*d6*, ppm) *δ* 7.15-7.16 (2d, 2H, *J* = 7.1 Hz, 2CH, pyridazine),7.76-7.77 (2d, 2H, *J* = 7.0 Hz, 2CH, pyrimidine), 9.15 (s, NH, D_2_O exchangeable); 10.10 (s, NH, D_2_O exchangeable); ^13^C NMR (DMSO-*d*_6_) *δ* 114.6, 122.8, 130.9, 147.5, 149.1, (5C, Ar-C),152.6, 163.4, 176.6 (3C, three imidic carbonyl groups); MS (70 ev, %): *m*/*z* = 206 (M^+^, 100%); Anal. Calc. for C_8_H_6_N_4_O_3_ (206.16): C, 46.61 (46.55); H, 2.93 (2.99); N, 27.18 (27.28).

### 3.3. Synthesis of 8-Methyl-2H,6H-pyridazino[1,2-a] pyrimido[2,1-c] [1,2,4]triazepine-2,6,10,13-tetraone (**4**)

A solution was prepared by combining 2.06 g (10 mmol) of compound (**3)** with 1.27 mL (10 mmol) of ethyl acetoacetate in a sodium ethoxide solution. This sodium ethoxide solution was made by dissolving 0.23 g of sodium metal in 35 mL of ethanol. The mixture was heated under reflux with stirring for 15 to 18 h, and progress was monitored by thin-layer chromatography (TLC). After the reaction was complete, the mixture was cooled and poured into 100 mL of cold water. It was then neutralized with acetic acid, which caused a solid to precipitate. This solid was filtered out and subsequently crystallized from methanol in good yield (85%), yellowish crystals and a melting point >350 °C (melted); IR (*ν*, cm^−1^) KBr; 3100 (CH aryl), 2980 (CH alkyl),1690, 1685, 1680, 1670 (4CO, four imidic carbonyl group), 1637(C=N), 1592 (C=C); ^1^H NMR (DMSO-*d6*, ppm) *δ* 2.10 (s, 3H, CH_3_), 5.20 (s, 1H, triazepine ring), 5.30 (d, 1H, *J* = 6.8 Hz, CH, pyrimidine), 7.20 (d, 1H, *J* = 6.9 Hz, CH, pyrimidine), 7.30 (d, 1H, *J* = 7.0 Hz, CH, pyridazine), 7.80 (d, 1H, *J* = 7.1 Hz, CH, pyridazine; ^13^C NMR (DMSO-*d*_6_) *δ* 19.4 (1C, CH_3_), 109.9 (1C, CH, triazepine ring), 114.6, 122.8, 130.9, 147.5, 149.2 (6C, Ar-C),152.6, 153.6, 163.4, 174.7 (4C, four imidic carbonyl groups); MS (70 ev, %): *m*/*z* = 272 (M^+^, 100%); Anal. Calc. for C_12_H_8_N_4_O_4_ (272.22): C, 52.95 (52.85); H, 2.96 (2.90); N, 20.58 (20.65).

### 3.4. Synthesis of 2H,6H-Pyridazino[1,2-a] pyrimido[2,1-c] [1,2,4]triazepine-2,6,8,10,13(7H)-pentaone (**5**)

**Method A**: First, prepare a warmed ethanolic sodium hydroxide solution by dissolving 10 mmol of NaOH in 50 mL of ethanol. Next, add **3** (2.06 g, 10 mmol) to the solution and heat the mixture for 60 min. After heating, allow the mixture to cool to room temperature. Then, add either 1.5 mL of diethyl malonate (10 mmol) or 1.04 g of malonic acid (10 mmol). Stir the mixture under reflux for 5 to 7 h, periodically monitoring the reaction using thin-layer chromatography (TLC). Once the response is complete, cool the mixture to room temperature and pour it into 100 mL of cold water. The solid product that forms should be filtered off, washed with 100 mL of water, and then dried. Finally, crystallize the product from an appropriate solvent.

**Method B**: A mixture of compound **3** (2.06 g, 10 mmol) and the corresponding diketone, such as diethyl malonate (1.5 mL, 10 mmol) or malonic acid (1.04 g, 10 mmol), was stirred under reflux in absolute ethanol (40 mL) for 8–10 h, with monitoring by thin-layer chromatography (TLC). After the reaction was complete, the mixture was cooled to 0 °C for 3–4 h. The resulting precipitate was filtered, dried, and crystallized from an appropriate solvent, such as ethanol, yielding brownish crystals compound **(5)** in high yield (82%) and with a melting point >350 °C (melted); IR (*ν*, cm^−1^) KBr; 3120 (CH aryl), 2977 (CH alkyl),1700, 1692, 1686, 1681, 1677 (5CO, five imidic carbonyl group), 1635 (C=N), 1590 (C=C); ^1^H NMR (DMSO-*d6*, ppm) *δ* 3.72 (s, 2H, CH_2,_ triazepine ring), 5.21 (d, 1H, *J* = 6.8 Hz, CH, pyrimidine), 6.89 (d, 1H, *J* = 6.90 Hz, CH, pyrimidine), 7.16 (d, 1H, *J* = 7.10 Hz, CH, pyridazine), 7.76 (d, 1H, *J* = 7.20 Hz, CH, pyridazine; ^13^C NMR (DMSO-*d_6_*) *δ* 30.8 (1C, CH_2,_ triazepine ring), 115.7, 128.9, 140.9, 148.7 (5C, Ar-C),152.6, 160.4, 161.0, 162.6, 172.9 (5C, five imidic carbonyl groups); MS (70 ev, %): *m*/*z* = 274 (M^+^, 100%); Anal. Calc. for C_11_H_6_N_4_O_5_ (274.19): C, 48.19 (48.25); H, 2.21 (2.30); N, 20.43(20.35).

### 3.5. Synthesis of 8-Hydroxy-6,8-dimethyl-2H,8H-pyridazino[1,2-a] pyrimido[2,1-c] [1,2,4]triazepine-2,10,13-trione (**6**)

A mixture of compound 3 (2.06 g, 10 mmol) and acetylacetone (1 mL, 10 mmol) was heated under reflux for 14–17 h, with the reaction progress monitored by thin-layer chromatography (TLC). The reaction was conducted in 30 mL of dry xylene, to which five drops of piperidine were added. After cooling, a solid precipitate formed. This precipitate was poured into 100 mL of water, filtered, and washed with 30 mL of ethanol. The obtained solid was then crystallized from dioxane, resulting in yellowish crystals **(6)** with a high yield of 85% and a melting point >350 °C (melted); IR (*ν*, cm^−1^) KBr; 3510 (brs, OH), 3130 (CH aryl), 2980 (CH alkyl),1688, 1684, 1673 (3CO, three imidic carbonyl group), 1632 (C=N), 1593 (C=C); ^1^H NMR (DMSO-*d6*, ppm) *δ* 1.18 (s, 3H, CH_3_), 2.24 (s, 3H, CH_3_), 4.23 (s, 1H, CH_,_ triazepine ring), 5.80 (d, 1H, *J* = 7.0 Hz, CH, pyrimidine), 7.92 (d, 1H, *J* = 7.1 Hz, CH, pyrimidine), 8.20 (d, 1H, *J* = 7.01 Hz, CH, pyridazine), 8.22 (d, 1H, *J* = 7.05 Hz, CH, pyridazine),11.52 (br., OH, D_2_O exchangeable); ^13^C NMR (DMSO-*d_6_*) *δ* 19.5, 23.7 (2C, 2CH_3_), 80.6 (1C_,_ triazepine ring), 104.5 (1C_,_ CH, triazepine ring), 112.0, 130.2, 130.3, 149.1, 152.1 (6C, Ar-C), 160.7, 160.8, 171.2 (3C, three imidic carbonyl groups); MS (70 ev, %): *m*/*z* = 288 (M^+^, 100%); Anal. Calc. for C_13_H_12_N_4_O_4_ (288.26): C, 54.17 (54.10); H, 4.20 (4.28); N, 19.44(19.52).

### 3.6. Synthesis of 8-(Chloromethylene)-6-methyl-2H,8H-pyridazino[1,2-a] pyrimido[2,1-c][1,2,4]triazepine-2, 10, 13-trione (**7**)

A solution of compound 3 (2.06 g, 10 mmol) was prepared by mixing it with a diketone, such as chloroacetylacetone (1.13 mL, 10 mmol), in a sodium ethoxide solution (prepared by dissolving 0.23 g of sodium metal in 40 mL of ethanol). This mixture was stirred under reflux for 12 to 15 h, with monitoring using thin-layer chromatography (TLC). After the reaction was complete, the mixture was cooled to 0 °C for 2 to 2 h. The resulting precipitate was then filtered, dried, and crystallized from dimethyl-formamide, resulting in the formation of yellowish crystals (product **7**) with a high yield of 80%. The melting point of the product is >350 °C (melted); IR (*ν*, cm^−1^) KBr; 3110 (CH aryl), 2988 (CH alkyl),1691, 1686, 1675 (3CO, three imidic carbonyl group), 1628 (C=N), 1591 (C=C); 1H NMR (DMSO-*d6*, ppm) *δ* 1.22 (s, 3H, CH3), 3.96 (s, 1H, CH, triazepine ring), 5.10 (s, 1H, CH-Cl), 5.71 (d, 1H, *J* = 7.1 Hz, CH, pyrimidine), 7.09 (d, 1H, *J* = 7.07 Hz, CH, pyrimidine), 7.11 (d, 1H, *J* = 7.1 Hz, CH, pyridazine), 7.86 (d, 1H, *J* = 7.08 Hz, CH, pyridazine); ^13^C NMR (DMSO-*d6*) *δ* 24.8 (1C, CH_3_), 95.1 (1C, CH-Cl), 98.7 (1C, CH, triazepine ring), 111.5, 130.8, 136.7, 139.1, 150.3, 152.9 (7C, Ar-C), 156.8, 160.1, 170.5 (3C, three imidic carbonyl groups); MS (70 ev, %): *m*/*z* = 304 (M^+^, 100%); Anal. Calc. for C_13_H_9_ClN_4_O_3_ (304.69): C, 51.25 (51.18); H, 2.98 (2.92); N, 18.39 (18.45).

### 3.7. Synthesis of 6,8-Diethylidene-2H,6H,8H-pyridazino[1,2-c]pyrimido[2,1-e][1,3,4,6]oxatriazepine-2,10,13-trione (**8**)

A reflux reaction involving compound **3** (2.06 g, 10 mmol) and propionic anhydride (1.30 mL, 10 mmol) was conducted in an ethanolic sodium ethoxide solution prepared by dissolving 0.23 g of sodium metal in 45 mL of ethanol. This reaction was maintained for 18 to 20 h with monitoring using thin-layer chromatography (TLC). After the reaction was complete, the mixture was allowed to cool and then poured into a mixture of water and ice. The mixture was then acidified with concentrated hydrochloric acid (HCl). The resulting precipitate was filtered, washed with water, dried, and recrystallized from dioxane, yielding yellowish crystals with a 77% yield. The melting point of the product is observed to be greater than >350 °C (melted); IR (*ν*, cm^−1^) KBr; 3138 (CH aryl), 2970 (CH alkyl),1696,1684,1671 (3CO, three imidic carbonyl group), 1625 (C=N), 1584 (C=C); ^1^H NMR (DMSO-*d6*, ppm) *δ* 3.13 (s, 6H, 2CH_3_), 3.81(s, 1H, C=CH), 4.12 (s, 1H, C=CH), 5.65 (d, 1H, *J* = 6.95 Hz, CH, pyrimidine), 6.89 (d, 1H, *J* = 7.01 Hz, CH, pyrimidine), 7.04 (d, 1H, *J* = 7.05 Hz, CH, pyridazine), 7.45 (d, 1H, *J* = 7.10 Hz, CH, pyridazine); ^13^C NMR (DMSO-*d*_6_) *δ* 21.2, 21.3 (2C, 2CH_3_), 101.4 (1C, C=CH),107.7 (1C, C=CH), 126.1, 126.8, 129.6, 134.1, 138.4, 139.3 (7C, Ar-C), 152.2, 156.2, 174.2 (3C, three imidic carbonyl groups); MS (70 ev, %): *m*/*z* = 300 (M^+^, 100%); Anal. Calc. for C_14_H_12_N_4_O_4_ (300.27): C, 56.00 (56.10); H, 4.03 (4.10); N, 18.66 (18.60).

### 3.8. Synthesis of 8-Amino-2H,6H-pyridazino[1,2-a]pyrimido[2,1-c][1,2,4]triazepine-2,6,10,13-tetraone (**9**)

To a warmed solution of sodium ethoxide in ethanol (prepared by dissolving 0.23 g of sodium metal, equivalent to 10 mmol, in 40 mL of absolute ethanol), compounds **3** (2.06 g, 10 mmol) and either ethyl cyanoacetate (1.10 mL, 10 mmol) or cyanoacetic acid (0.85 g, 10 mmol) were added. The mixture was stirred under reflux for 19 to 22 h. After the reaction was complete, the mixture was cooled to room temperature and then poured into 100 mL of cold water. This was followed by neutralization with acetic acid. The resulting solid product precipitated out, was filtered off, washed with water and ethanol, dried, and then crystallized from dimethylformamide, resulting in yellow crystals with an 79% yield. The melting point of the product was observed to be>350 °C. IR (*ν*, cm^−1^) KBr; 3425 (brs, NH_2_), 3155 (CH aryl), 2920 (CH alkyl), 1698,1690, 1684,1677 (4CO, four imidic carbonyl groups), 1634 (C=N), 1593(C=C); ^1^H NMR (DMSO-*d6*, ppm) *δ* 4.07 (s, 1H, CH, triazepine ring), 5.39 (d, 1H, *J* = 7.05 Hz, CH, pyrimidine), 7.25 (br., 2H, NH_2_, D_2_O exchangeable) 7.30 (d, 1H, *J* = 7.03 Hz, CH, pyrimidine), 7.31 (d, 1H, *J* = 7.00 Hz, CH, pyridazine), 7.88 (d, 1H, *J* = 7.08 Hz, CH, pyridazine); ^13^C NMR (DMSO-*d*_6_) *δ* 93.8 (1C, CH, triazepine ring),114.7, 129.7, 129.9, 133.9, 138.4 (6C, Ar-C), 152.1, 160.5, 167.2, 171.3 (4C, four imidic carbonyl groups); MS (70 ev, %): *m*/*z* = 273 (M^+^, 100%); Anal. Calc. for C_11_H_7_N_5_O_4_ (273.21): C, 48.36 (48.30); H, 2.58 (2.65); N, 25.63 (25.70).

### 3.9. Synthesis of 6-Amino-8-imino-2H,8H-pyridazino[1,2-a]pyrimido[2,1-c][1,2,4]triazepine-2,10,13-trione (**10**)

A mixture of compound 3 (2.06 g, 10 mmol) and malononitrile (0.6 mL, 10 mmol) was prepared in 50 mL of ethanol containing 1 mL of piperidine as a catalyst. The mixture was heated under reflux for 30 to 35 h, with reaction progress monitored using thin-layer chromatography (TLC). After heating, the reaction mixture was cooled and poured into crushed ice, then acidified with hydrochloric acid. The resulting solid was filtered off, washed with water, dried, and recrystallized from dioxane, yielding yellowish crystals with a yield of 72%. The melting point of the product was greater than >350 °C (melted). IR spectroscopy was performed, with the measurements taken in KBr, IR (*ν*, cm^−1^) KBr; 3430 (brs, NH_2_), 3350 (brs, NH), 3145 (CH aryl), 2915 (CH alkyl),1700,1695, 1682 (3CO, three imidic carbonyl group), 1631 (C=N), 1590 (C=C); ^1^H NMR (DMSO-*d6*, ppm) *δ* 3.78 (s, 1H, CH, triazepine ring), 5.50 (d, 1H, *J* = 7.01 Hz, CH, pyrimidine), 6.10 (br., 2H, NH_2_, D_2_O exchangeable), 7.11 (d, 1H, *J* = 7.04 Hz, CH, pyrimidine), 7.80 (d, 1H, *J* = 6.92 Hz, CH, pyridazine), 7.86 (d, 1H, *J* = 6.98 Hz, CH, pyridazine), 9.22 (br.,1H, NH, D_2_O exchangeable); ^13^C NMR (DMSO-*d*_6_) *δ* 71.8 (1C, CH, triazepine ring), 127.0, 127.6, 128.4, 129.2, 140.0, 151.5 (7C, Ar-C), 156.2, 160.6, 175.01 (3C, three imidic carbonyl groups); MS (70 ev, %): *m*/*z* = 272 (M^+^, 100%); Anal. Calc. for C_11_H_8_N_6_O_3_ (272.22): C, 48.53 (48.60); H, 2.96 (2.90); N, 30.87 (30.80).

### 3.10. Biological Screening (Materials and Methods, In Vitro)

The antimicrobial activity of the prepared compounds was tested in vitro against various types of bacteria and fungi. The tested organisms included Gram-negative bacteria (*Klebsiella pneumoniae* and *Escherichia coli*), Gram-positive bacteria (*Streptococcus pyogenes* and *Staphylococcus aureus*), and fungi (*Candida albicans*, *Curvularia lunata*, *Alternaria alternata*, and *Aspergillus niger*). The newly prepared compounds were dissolved in dimethyl sulfoxide (DMSO) and assessed for antimicrobial activity using the agar disk diffusion method. Cefotaxime sodium and nystatin [5,53,55,56,59,66,67,68,69,70,71], were used as standards for the antibacterial and antifungal assays, respectively. A solution of each tested compound at a concentration of 100 μg/mL was prepared, and microplate wells measuring 1 cm in diameter were utilized. The zones of inhibition were measured using calipers or automated scanners and compared to those of the standard drugs. Specifically, cefotaxime sodium (0.15 μmol/mL) was used for antibacterial activity, while nystatin (0.037 μmol/mL) served for antifungal activity. Compound-impregnated disks were placed on an agar plate containing a standardized microbial suspension, followed by incubation at 37 °C for 24 h. To determine the minimum inhibitory concentration (MIC) using the serial plate dilution method [5,53,55,56,59,81,82,83,84,85,86], 5 mg of each tested compound was dissolved in 1 mL of DMSO to create stock solutions. Serial dilutions were then performed from each stock solution, and the plates were incubated at 37 °C for 24 h. The MIC is defined as the lowest concentration (μmol/mL) of the tested compound that prevents visible growth on the plates. DMSO served as a solvent control to confirm that it did not affect bacterial growth. The assay results are presented in Table 1 and Table 2.

### 3.11. The Sources of Strain for Different Types of Bacteria and Fungi

The researchers at the Department of Chemistry of Natural and Microbial Products at the National Research Centre in Giza, Egypt, prepared various strains of bacteria and fungi based on previous studies. The strains include Gram-negative bacteria such as *Klebsiella pneumoniae* and *Escherichia coli*, Gram-positive bacteria such as *Streptococcus pyogenes* and *Staphylococcus aureus*, and fungi such as *Candida albicans*, *Curvularia lunata*, *Alternaria alternata*, and *Aspergillus niger*.

### 3.12. Klebsiella pneumonia (G-ve)

*Klebsiella pneumoniae* is a Gram-negative, non-motile bacterium that is encapsulated and commonly found in the environment. It has been associated with pneumonia, particularly in patients who consume alcohol or have diabetes mellitus. This bacterium typically colonizes the mucosal surfaces of the gastrointestinal and oropharyngeal tracts in humans [90].

### 3.13. Escherichia coli (G-ve)

*E. coli* is a rod-shaped, Gram-negative bacterium that belongs to the Enterobacteriaceae family. Laboratory strains of E. coli are non-pathogenic microorganisms that grow rapidly in a variety of solid or liquid media, especially in the presence of oxygen, with a doubling time of approximately 20 min. They can also thrive in anaerobic conditions, making them facultative anaerobes. The genotypes and phenotypes of common laboratory strains of E. coli have been extensively studied, leading to their use as a model organism for teaching and research over many decades [91].

### 3.14. Streptococcus pyogenes (G + ve)

The role of *S. pyogenes* biofilm in cellulitis was investigated using a murine model for soft-tissue infection [92].

### 3.15. Staphylococcus aureus(G + ve)

*S. aureus* is commonly found in the environment and as part of the normal human flora, particularly on the skin and mucous membranes, often in the nasal area of healthy individuals. Usually, S. aureus does not cause infections on healthy skin. However, if it enters the bloodstream or penetrates tissues, it can lead to various potentially serious infections [93].

### 3.16. The Fungi of Candida albicans

The yeast *Candida albicans* is a part of the microbiota found in the oral, gastrointestinal, and genital tracts of healthy individuals. The prevalence of Candida on the mucosal surfaces in these individuals ranges from 30% to 70% [94].

### 3.17. The Fungi of Curvularia lunata

*Curvularia lunata* is a genus of ascomycete fungi that primarily includes saprotrophic and plant pathogenic species. It is regarded as one of the most significant groups of filamentous fungi associated with both local and invasive phaeohyphomycoses and with human infections caused by dematiaceous fungi [95].

### 3.18. The Fungi of Alternaria alternate

The obligate biotrophic fungus Puccinia striiformis f. sp. tritici (Pst) is responsible for causing stripe (yellow) rust on wheat worldwide. Notably, a specific strain of fungus has been found to hyper-parasitize Pst. This strain was isolated from gray-colored rust pustules and identified as *Alternaria alternata* based on a combination of morphological characteristics [96].

### 3.19. The Fungi of Aspergillus niger

Some strains of *Aspergillus niger* produce sclerotia under specific conditions. Sclerotia are clusters of hyphae that can function as survival or reproductive structures in species related to A. niger [97].

### 3.20. Computational Methods: Molecular Docking of Synthesized Compounds

All protein receptors were obtained from the RCSB database. The target protein structures were preprocessed in PyMOL software (v2.5), including the removal of water molecules, ions, and any pre-existing ligands. The structures of the compounds were created using BIOVIA Draw [98]. Subsequently, Open Babel [99] was used to convert each compound to the mol2 format. Next, Autodock tools were used to convert the molecules to PDBQT format. Before docking, ligand-centred maps were generated using AutoDock [100]. The 2-D interactions between the targets and ligands were analyzed using the BIOVIA Discovery Studio software program version 17.2 (BIOVIA, 2017), [68,98]. The accuracy was evaluated by calculating the Root Mean Square Deviation (RMSD) between the docked and crystallographic poses. The RMSD values ranged from 0.99 Å to 1.59 Å.

#### 3.20.1. Ethical Approval and Consent to Participate

This study did not involve human or animal subjects. However, all procedures were conducted with approval from the Medical Research Ethics Committee of the National Research Centre, Department of Chemistry of Natural and Microbial Products, Giza 12622, Egypt.

#### 3.20.2. Human and Animal Rights

No human or animal subjects were used in this study. The research was conducted in accordance with ethical standards for in vitro research.

#### 3.20.3. Chemicals and Drugs

The study included various microorganisms: Gram-positive bacteria, such as *Staphylococcus aureus* and *Streptococcus pyogenes*; Gram-negative bacteria, such as *Escherichia coli* and *Klebsiella pneumoniae*; and fungi, such as *Aspergillus niger*, *Alternaria alternata*, *Curvularia lunata*, and *Candida albicans*. These were sourced from the National Research Centre, Department of Chemistry of Natural and Microbial Products, located in Giza, Egypt. Additionally, cefotaxime sodium, nystatin, and dimethyl sulfoxide (DMSO) were obtained from Sigma-Aldrich.

## 4. Conclusions

In this study, we synthesized several new compounds starting from 1-(4-oxo-1,4-dihydropyrimidin-2-yl)-1,2-dihydropyridazine-3,6-dione. Among these compounds are the promising derivatives pyridazinopyrimido[2,1-*c*][1,2,4]triazepine and pyridazino[1,2-*c*] pyrimido[2,1-*e*][1,3,4,6]oxatriazepine, which were obtained in excellent yields using novel, rapid, and effective methods. Biological evaluations demonstrated that some of these compounds, compounds explicitly **6**, **8**, **9**, and **10**, exhibit encouraging antimicrobial activities. These compounds feature various functional groups, including pyridazino-pyrimidine, 1,2,4-triazepine, 1,3,4,6-oxatriazepine, amine, imine, ethylidene, hydroxy, and methyl groups. When compared to standard drugs such as cefotaxime sodium and nystatin, their activity is noteworthy. This study concludes that compounds **6**, **8**, **9**, and **10** show exceptional potential as broad-spectrum antimicrobial agents, supported by consistent in silico evidence. They demonstrate potent, high-affinity binding to a diverse range of essential bacterial and fungal targets, including DNA gyrase, a virulence-associated sortase, a drug-resistant carbapenemase, and key fungal enzymes involved in sterol synthesis and pathogenicity. Their predicted inhibitory activity consistently exceeds that of standard reference drugs. The binding mechanisms are structurally rationalized through critical interactions, particularly hydrogen bonds with catalytic residues and extensive stabilizing hydrophobic networks within the active sites of all targeted enzymes. These robust computational results validate these compounds as prime multi-target leads, underscoring the urgent need to advance them into in vitro and in vivo experimental studies for the development of novel therapeutic agents.

## Figures and Tables

**Table 1:** The minimum inhibitory concentrations of the compounds against bacteria.

MIC (µmol mL^−1^)				
Compounds	Microorganisms			
	Gram-Negative Bacteria		Gram-Positive Bacteria	
	*Klebsiella pneumoniae*	*E. coli*	*Streptococcus pyogenes*	*Staphylococcus aureus*
**1**	21	23	25	27
**2**	18	20	22	24
**3**	13	15	16	17
**4**	12	14	15	16
**5**	8	7	8	9
**6**	4	3	3	4
**7**	5	4	4	5
**8**	3	2	2	3
**9**	2	2	1	2
**10**	1	1	1	2
Cefotaxime sodium	1	1	1	2
Negative control	NI	NI	NI	NI

DMSO was used as the negative control and as the solvent for test compounds and the reference drug.

**Table 2:** The minimum inhibitory concentrations of the compounds against fungi.

MIC (µmol mL^−1^)				
Compounds	Microorganisms			
	*Candida albicans*	*Curvularia lunata*	*Alternaria alternata*	*Aspergillus niger*
**1**	26	29	30	31
**2**	20	22	25	28
**3**	15	17	18	19
**4**	10	12	13	14
**5**	7	8	9	10
**6**	5	6	6	7
**7**	6	7	8	9
**8**	3	4	4	5
**9**	2	2	3	4
**10**	1	1	2	3
Nystatin	1	1	2	3
Negative control	NI	NI	NI	NI

DMSO was used as the negative control and as the solvent for test compounds and the reference drug.

**Table 3:** Molecular interactions of ligands with amino acids of Gyrase B of *Staphylococcus aureus* (PDB: ID 4URM).

	Protein	Ligand	3D Structure	Hydrophilic Interactions	Hydrophobic Contacts			No. of H-Bonds	No. of Total Bonds	Affinity kcal mol^−1^
				Residue (H-Bond)	Length	Residue (Bond Type)	Length			
1	GyraseB of *B. pumilis* (PDB: ID 4URM)	6	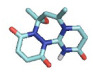	Lys203, (H- Bond) Arg239, (H- Bond)	2.02 2.16	His55, (Pi–alkyl) Lys203, (Pi–alkyl) Arg239, (Pi–Cation) Phe172, (Pi–Pi Stacked)	2.82 5.20 3.54 5.21	**2**	6	**−9.30**
2		8	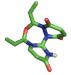	Arg52, (H- Bond) Arg239, (H- Bond) His241, (H- Bond)	2.47 3.09 2.93	Val49, (Carbon H. bond)	3.55	**3**	4	**−9.30**
3		9	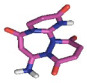	Arg239, (H- Bond) Asn11, (H- Bond) Asn11, (H- Bond) His241, (H- Bond) Arg52, (H- Bond) Ser50, (H- Bond)	2.19 2.86 2.25 2.09 2.24 2.75	Lys203, (Carbon H. bond)	3.45	**6**	7	**−9.20**
		10	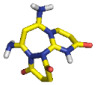	His241, (H- Bond) Ser50, (H- Bond) Val49, (H- Bond) Lys203, (H- Bond) Asp84, (H- Bond)	3.05 2.58 2.97 2.12	Asp84, (Pi–cation) Asp84, (Carbon H. bond)	5.46 3.53	**5**	7	**−9.10**
4		Cefotaxime	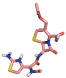	Asn103, (H- Bond) Val49, (H- Bond) Arg239, (H- Bond) Arg202, (H- Bond)	2.13 2.34 3.01 2.52	Ile86, (Pi–sigma) Asp54, (Pi–Pi Stacked)	3.58 4.72	1	3	−7.10

**Table 4:** Molecular interactions of ligands with amino acids of DNA Gyrase of *E. coli* (PDB: ID 7P2M).

NO	Protein	Ligand	3D Structure	Hydrophilic Interactions		Hydrophobic Contacts		No. of H-Bonds	No. of Total Bonds	Affinity kcal mol^−1^
				Residue (H-Bond)	Length	Residue (Bond Type)	Length			
1	DNA Gyrase of *E. coli* (PDB: ID 7P2M)	6	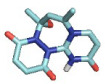	Thr165, (H- Bond) Gly77, (H- Bond) Asn46, (H- Bond)	3.32 2.99 2.45	Arg76, (Pi–Cation) Val120, (alkyl) Ile78, (Pi–alkyl) Ile78, (Pi–alkyl) Ile94, (Pi–alkyl)	4.07 4.96 5.06 2.66 4.69	**3**	8	−7.60
2		8	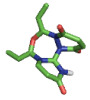	-	-	Val120, (alkyl) Ile78, (Pi–alkyl) Ile78, (Pi–alkyl) Arg76, (Pi–Cation) Glu50, (Pi–Cation)	4.25 4.94 5.73 4.34 4.11	**0**	5	−7.40
3		9	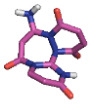	Arg76, (H- Bond) Thr165, (H- Bond)	3.20 3.30	Ile78, (Pi–Cation) Glu50, (Pi–Cation) Arg76, (alkyl)	5.01 3.55 4.29	**2**	10	−7.80
4		10	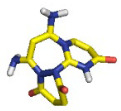	-	-	Glu50, (Pi–cation) Glu50, (Pi–cation) Asp73, (Pi–Cation) Asn46, (Carbon–H bond)	4.77 5.10 2.89 2.86	**0**	4	−7.30
5		Cefotaxime	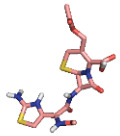	Ala47, (H- Bond) Asn46, (H- Bond) Ser121, (H- Bond) Ile94, (H-Bond)	2.75 2.40 2.64 1.95	Ile78, (alkyl) Val120, (alkyl) Asp73, (Carbon–H bond)	4.25 5.13 3.41	4	7	−7.10

**Table 5:** Molecular interactions of ligands with amino acids of Sortase A from *Streptococcus pyogenes* (PDB: ID 8T8G).

No	Protein	Ligand	3D Structure	Hydrophilic Interactions		Hydrophobic Contacts		No. of H-Bonds	No. of Total Bonds	Affinity kcal mol^−1^
				Residue (H-Bond)	Length	Residue (Bond Type)	Length			
1	Sortase A from *Streptococcus pyogenes* (PDB: ID 8T8G)	6	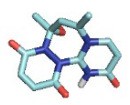	-	-	Val206, (alkyl) Arg216, (alkyl) Leu113, (Pi–alkyl) Val191, (Carbon–H bond) Ala140, (alkyl) Met125, (alkyl) Val191, (Pi–sigma) Ile194,(alkyl)	5.04 5.29 4.49 3.93 4.93 3.41 5.78 3.85	0	8	−6.40
2		8	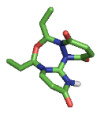	-	-	Val186, (alkyl) Val206, (alkyl) Val193, (Pi–alkyl) Pro188, (Carbon–H bond) Ile218, (alkyl) Ile194, (alkyl) Met125, (alkyl) Val191, (alkyl)	4.80 4.68 4.11 5.45 4.06 5.27 4.01 3.37	0	8	−6.80
		9	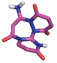	Val191, (H- Bond) Pro188, (H- Bond) Arg216, (H- Bond)	2.88 2.70 1.38	-	-	3	3	−6.10
		10	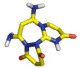	Pro188, (H- Bond)	2.53	-	-	1	1	−6.00
6		Cefotaxime	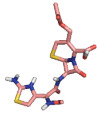	Arg216, (H- Bond) Pro188, (H- Bond) Met125, (H- Bond) Met125, (H- Bond) Thr124, (H- Bond)	2.47 2.16 1.87 2.66 2.25	Leu113, (Pi–sigma)	3.48	5	6	−6.10

**Table 6:** Molecular interactions with amino acids of KPC-2 carbapenemase of *K. pneumoniae* (PDB: ID 2OV5).

	Protein	Ligand	3D Structure	Hydrophilic Interactions	Hydrophobic Contacts			No. of H-Bonds	No. of Total Bonds	Affinity kcal mol^−1^
				Residue (H-Bond)	Length	Residue (Bond Type)	Length			
1	KPC-2 carbapenemase of *K. pneumoniae* (PDB: ID 2OV5)	6	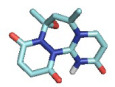	Ser130 (H- Bond) Asn170 (H- Bond)	2.67 2.58	Trp105, (Pi–Pi shaped) Trp105, (Pi–alkyl) Trp105, (CH-bond) Asn132, (CH-bond) Leu167, (Pi–alkyl)	3.93 5.27 3.43 3.17 5.47	2	7	−8.10
2		8	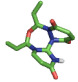	Lys234, (H- Bond) Thr216, (H- Bond) Arg220, (H- Bond) Thr237, (H- Bond)	2.48 2.22 2.56 1.89	Trp105, (Pi–alkyl) His274, (Pi–alkyl) Ser130, (CH-bond)	4.46 5.10 2.97	4	7	−7.90
3		9	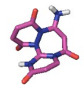	Thr237, (H- Bond) Lys73, (H- Bond) Asn132, (H- Bond) Glu166, (H- Bond)	2.70 2.80 2.25 2.88	Trp105, (Pi–Pi shaped)	5.24	4	10	−8.90
4		10	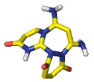	Ser70 (H- Bond)	2.07	Glu166, (Pi–cation) Glu166, (Pi–cation) Trp105, (CH-bond)	3.09 3.00 3.04	1	4	−7.30
5		Cefotaxime	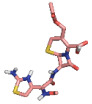	Lys73, (H- Bond) Ser70, (H- Bond) Thr237, (H- Bond) Thr235, (H- Bond) Thr215, (H- Bond)	2.57 2.69 2.77 2.85 2.62	Trp105, (Pi–alkyl) Trp105, (Sulfur) His219, (Pi–cation)	3.76 4.86 4.75	5	8	−7.40

**Table 7:** Molecular interactions of ligands with *A. niger* Fdc1 (PDB:ID 4ZA5).

NO	Protein	Ligand	3D Structure	Hydrophilic Interactions		Hydrophobic Contacts		No. of H-Bonds	No. of Total Bonds	Affinity kcal mol^−1^
				Residue (H- Bond)	Length	Residue (Bond Type)	Length			
1	*A. niger* Fdc1 (PDB:ID 4ZA5)	6	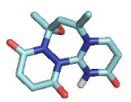	Ile171, (H-Bond) Ile171, (H-Bond) Gln190, (H-Bond)	2.00 2.77 2.24	Ala172, (Pi–alkyl) Met283, (Pi–alkyl) Leu439, (Pi–alkyl) Ile171, (Pi–alkyl) Ile327, (Pi–sigma)	4.96 4.39 5.20 3.78 3.65	3	8	−12.40
2		8	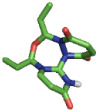	-	-	Ile327, (Pi–sigma) Ile327, (Pi–alkyl) Ile171, (Pi–alkyl) Phe280, (Pi–alkyl) Arg173, (Pi–alkyl) Ile171, (Carbon H bond)	3.62 4.56 3.85 5.18 3.96 3.64	0	6	−12.0
3		9	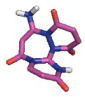	-	-	Thr323, (Pi–sigma) Ile171, (Pi–alkyl) Ile171, (Carbon H bond)	3.67 5.10 3.32	0	3	−9.00
4		10	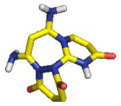	Ile171, (H-Bond) Ser223, (H-Bond)	2.13 2.00	-	-	2	2	−9.60
5		Nystatin	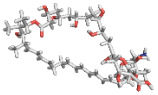	Trp169, (H-Bond) Met225, (H-Bond)	1.76 2.72	Ala172, (Pi–alkyl)	3.60	2	3	−8.10

**Table 8:** Interactions of ligands with amino acids of Sterol 14-alpha demethylase of *C. albicans* (PDB: ID 5TZ1).

No	Protein	Ligand	3D Structure	Hydrophilic Interactions		Hydrophobic Contacts		No. of H-Bonds	No. of Total Bonds	Affinity kcal mol^−1^
				Residue (H- Bond)	Length	Residue (Bond Type)	Length			
1	Sterol 14-alpha demethylase of *C. albicans* (PDB:ID 5TZ1)	6	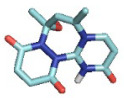	-	-	Tyr118, (Pi–alkyl) Ile379, (Pi–alkyl) Phe105, (Pi–alkyl) Ile379, (Pi–alkyl) His468, (Pi–alkyl) Leu376, (Pi–sigma) Cys470, (Sulfur)	3.63 4.97 5.18 5.44 5.47 4.95 4.92 4.20	0	7	−7.50
2		8	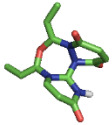	Arg381, (H-Bond) Lys143, (H-Bond)	2.53 3.00	Phe463, (Carbon H bond) Ile471, (Pi–alkyl) Cys470, (Pi–alkyl) Cys470, (Pi–alkyl) Phe463, (Pi–alkyl) His468, (Pi–alkyl)	3.19 5.14 4.64 3.91 5.43 2.11	2	8	−7.60
3		9	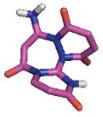	Leu376, (H-Bond) Thr311, (H-Bond)	1.99 2.96	Pro375, (Carbon H bond) Gly308, (Carbon H bond) Ile471, (Pi–alkyl) Ile304, (Pi–alkyl) Leu370, (Pi–alkyl) Ala476, (Pi–alkyl) Pro375, (Pi–alkyl) Thr311, (Pi–sigma)	3.00 3.32 4.92 5.24 4.82 4.13 4.95 3.86	2	10	−6.90
4		10	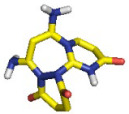	Cys470, (H-Bond) Gln479, (H-Bond)	2.96 2.83	Gly472, (Carbon H bond) Thr311, (Carbon H bond) Cys470, (Pi–alkyl) Ala476, (Pi–alkyl) Gly308, (Pi–sigma)	3.57 2.59 4.91 4.60 3.81	2	7	−6.90
5		Nystatin	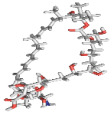	Tyr460, (H-Bond) Gly303, (H-Bond) Ile304, (H-Bond)	2.69 2.73 2.18	Pro462, (Carbon H bond) Leu461, (Carbon H bond) Ser312, (Carbon H bond) Tyr460, (Pi–alkyl) Trp427, (Pi–alkyl) Ile471, (Pi–alkyl) Ile304, (Pi–alkyl)	3.18 3.36 3.40 4.08 5.35 4.05 4.46	3	10	−6.20

**Table 9:** Interactions of ligands with amino acids of AaTPS of *Alternaria alternata* (PDB: ID 6LCD).

No	Protein	Ligand	3D Structure	Hydrophilic Interactions		Hydrophobic Contacts		No. of H-Bonds	No. of Total Bonds	Affinity kcal mol^−1^
				Residue (H-Bond)	Length	Residue (Bond Type)	Length			
1	AaTPS of *Alternaria alternata* (PDB:ID 6LCD)	6	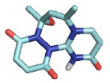	Asn392, (H-Bond) Asn307, (H-Bond) Val268, (H-Bond)	2.57 2.60 1.73	Phe149, (Pi–Pi Stacked) Phe149, (Pi–alkyl) Ile169, (Pi–alkyl) Ile153, (Pi–alkyl) Ile172, (Pi–alkyl) Ala269, (Pi–alkyl)	4.54 4.51 4.86 5.06 3.86 4.03	3	9	−8.40
2		8	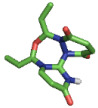	Asn307, (H-Bond) Asn307, (H-Bond) Arg264, (H-Bond)	2.33 2.77 2.68	Phe149, (Carbon H bond) Leu273, (Pi–alkyl) Tyr272, (Pi–alkyl) Ile172, (Pi–alkyl) Ile169, (Pi–alkyl)	3.26 4.77 4.56 3.94 5.10	3	8	−8.50
3		9	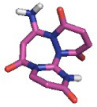	Asn307, (H-Bond) Asn307, (H-Bond) Val268, (H-Bond)	2.18 2.28 2.79	Phe146, (Pi–Pi Stacked) Phe149, (Pi–Pi Stacked)	4.80 4.85	3	5	−8.20
4		10	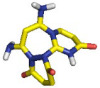	Asn307, (H-Bond) Asp267, (H-Bond)	2.15 2.52	Asp267, (Pi–cation) Asp176, (Pi–cation)	2.32 5.08	2	4	−8.30
5		Nystatin	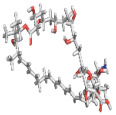	Lys314, (H-Bond) Ser311, (H-Bond) Tyr260, (H-Bond) Tyr260, (H-Bond) Asp267, (H-Bond) Asp367, (H-Bond) Tyr263, (H-Bond)	2.11 2.82 1.92 2.28 1.80 2.14 2.65	His136, (Carbon H bond) Tyr263, (Pi–alkyl)	3.01 5.44	7	9	−7.50

## Data Availability

The original contributions presented in this study are included in the article/Supplementary Materials. Further inquiries can be directed to the corresponding author.

## Supplementary Materials

The following supporting information can be downloaded at https://www.mdpi.com/article/10.3390/ph19010083/s1, Figure S1. ^1^H NMR Spectrum (500 MHz, DMSO-*d6*) of Compound **3**; Figure S2. ^13^C NMR Spectrum (125 MHz, DMSO-d6) of Compound **3**; Figure S3. 1H NMR Spectrum (500 MHz, DMSO-*d6*) of Compound **4**; Figure S4. ^13^C NMR Spectrum (125 MHz, DMSO-*d6*) of Compound **4**; Figure S5. ^1^H NMR Spectrum (500 MHz, DMSO-*d6*) of Compound **5**; Figure S6. ^13^C NMR Spectrum (125 MHz, DMSO-*d6*) of Compound **5**; Figure S7. ^1^H NMR Spectrum (500 MHz, DMSO-*d6*) of Compound **6**; Figure S8. ^13^C NMR Spectrum (125 MHz, DMSO-*d6*) of Compound **6**; Figure S9. ^1^H NMR Spectrum (500 MHz, DMSO-*d6*) of Compound **7**; Figure S10. ^1^H NMR Spectrum (500 MHz, DMSO-*d6*) of Compound **8**; Figure S11. ^13^C NMR Spectrum (125 MHz, DMSO-d6) of Compound **8**; Figure S12. ^1^H NMR Spectrum (500 MHz, DMSO-*d6*) of Compound **9**; Figure S13. ^13^C NMR Spectrum (125 MHz, DMSO-*d6*) of Compound **9**; Figure S14. ^1^H NMR Spectrum (500 MHz, DMSO-*d6*) of Compound **10**; Figure S15. ^13^C NMR Spectrum (125 MHz, DMSO-*d6*) of Compound **10**.
