# Supplementary material for: Design, Synthesis, Antimicrobial Activity and Molecular Docking of New 1,2,4-Triazepine, 1,3,4,6-Oxatriazepine and Pyridazino[1,2-a] Pyrimidine Derivatives
Source: Pharmaceuticals (Basel). 2025 Dec 31;19(1):83. doi: 10.3390/ph19010083 (PMC12845107; doi:10.3390/ph19010083)
Supplement: Supplementary file 1 [file pharmaceuticals-19-00083-s001.zip › pharmaceuticals-4072515-supplementary.pdf]

## Supplementary Material Data

# Design, Synthesis, Antimicrobial Activity and Molecular Docking of New 1,2,4-Triazepine, 1,3,4,6-Oxatriazepine and Pyridazino[1,2-*a*] Pyrimidine Derivatives

**Nasser Amri and Ameen Ali Abu-Hashem ***

Department of Physical Sciences, Chemistry Division, College of Science, Jazan University, P.O. Box 114, Jazan 45142, Saudi Arabia

* Correspondence: aminaliabuhashem@yahoo.com or aaabuhashem@jazanu.edu.sa;
Tel.: +966-0591363915 or +20-012-2521-1700; Fax: +966-73245212

| Table of Contents | Page |
|---|---|
| Figure S1 $^{1}H$ NMR Spectrum (500 MHz, DMSO-*d6*) of Compound **3** | 2 |
| Figure S2 13C NMR Spectrum (125 MHz, DMSO-d6) of Compound **3** | 3 |
| Figure S3 $^{1}H$ NMR Spectrum (500 MHz, DMSO-*d6*) of Compound **4** | 4 |
| Figure S4 $^{13}C$ NMR Spectrum (125 MHz, DMSO-*d6*) of Compound **4** | 5 |
| Figure S5 $^{1}H$ NMR Spectrum (500 MHz, DMSO-*d6*) of Compound **5** | 6 |
| Figure S6 $^{13}C$ NMR Spectrum (125 MHz, DMSO-*d6*) of Compound **5** | 7 |
| Figure S7 $^{1}H$ NMR Spectrum (500 MHz, DMSO-*d6*) of Compound **6** | 8 |
| Figure S8 $^{13}C$ NMR Spectrum (125 MHz, DMSO-*d6*) of Compound **6** | 9 |
| Figure S9 $^{1}H$ NMR Spectrum (500 MHz, DMSO-*d6*) of Compound **7** | 10 |
| Figure S10 $^{1}H$ NMR Spectrum (500 MHz, DMSO-*d6*) of Compound **8** | 11 |
| Figure S11 $^{13}C$ NMR Spectrum (125 MHz, DMSO-d6) of Compound **8** | 12 |
| Figure S12 $^{1}H$ NMR Spectrum (500 MHz, DMSO-*d6*) of Compound **9** | 13 |
| Figure S13 $^{13}C$ NMR Spectrum (125 MHz, DMSO-*d6*) of Compound **9** | 14 |
| Figure S14 $^{1}H$ NMR Spectrum (500 MHz, DMSO-*d6*) of Compound **10** | 15 |
| Figure S15 $^{13}C$ NMR Spectrum (125 MHz, DMSO-*d6*) of Compound **10** | 16 |

## $^1$H- NMR and $C^{13}$ - NMR spectra

*1-(4-oxo-1,4-dihydropyrimidin-2-yl)-1,2-dihydropyridazine-3,6-dione* (**3**)

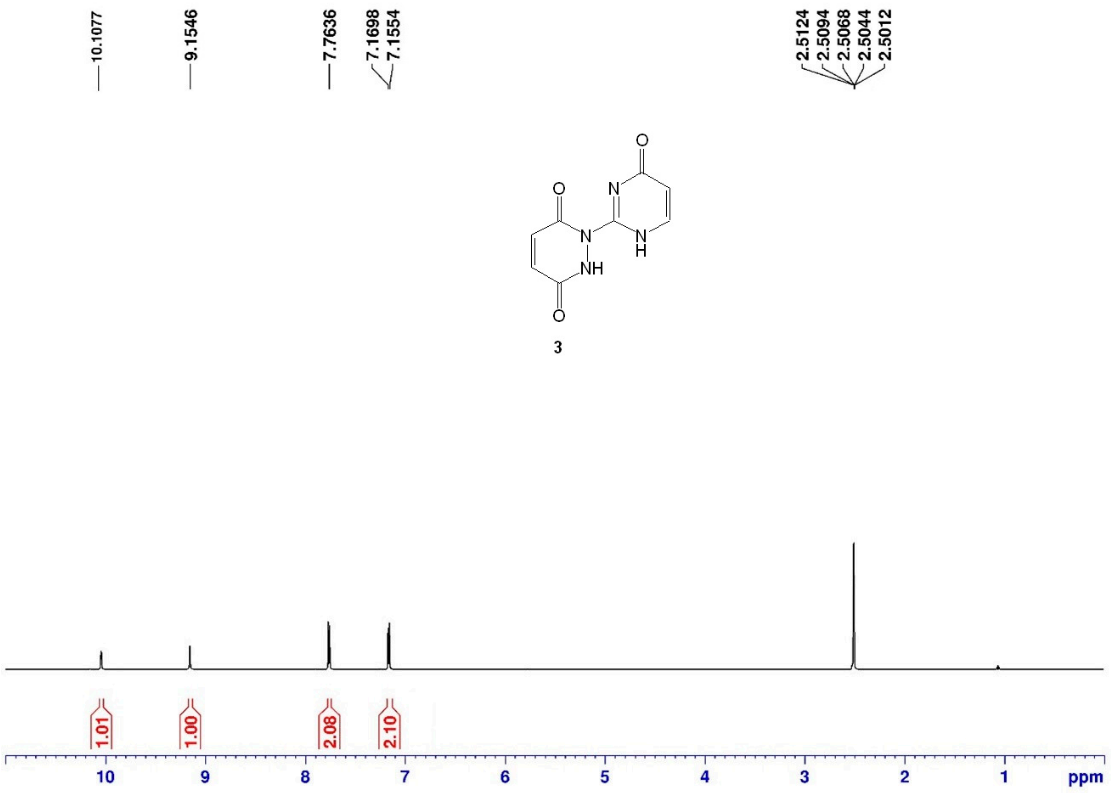


**Figure S1** $^1$H NMR Spectrum (500 MHz, DMSO-*d6*) of Compound **3**

***1-(4-oxo-1,4-dihydropyrimidin-2-yl)-1,2-dihydropyridazine-3,6-dione* (3)**

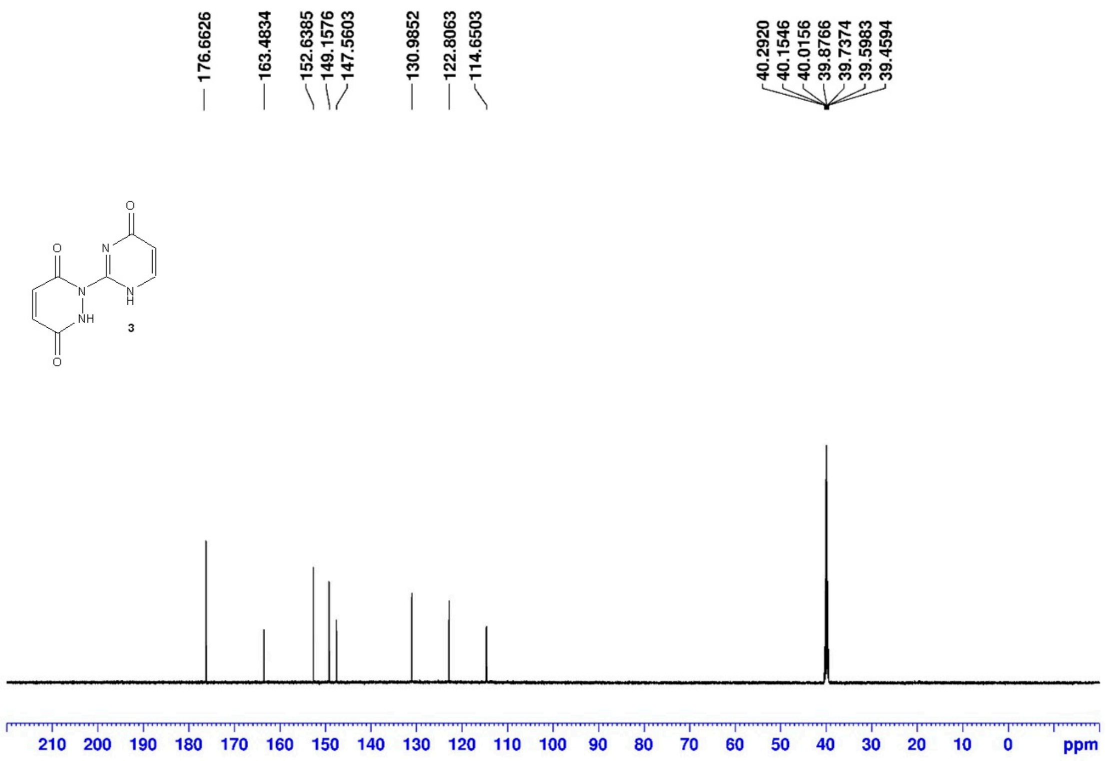


**Figure S2** $^{13}$C NMR Spectrum (125 MHz, DMSO-*d6*) of Compound **3**

***8-methyl-2H,6H-pyridazino[1,2-a] pyrimido[2,1-c] [1,2,4] triazepine-2,6,10,13-tetraone* (4)**

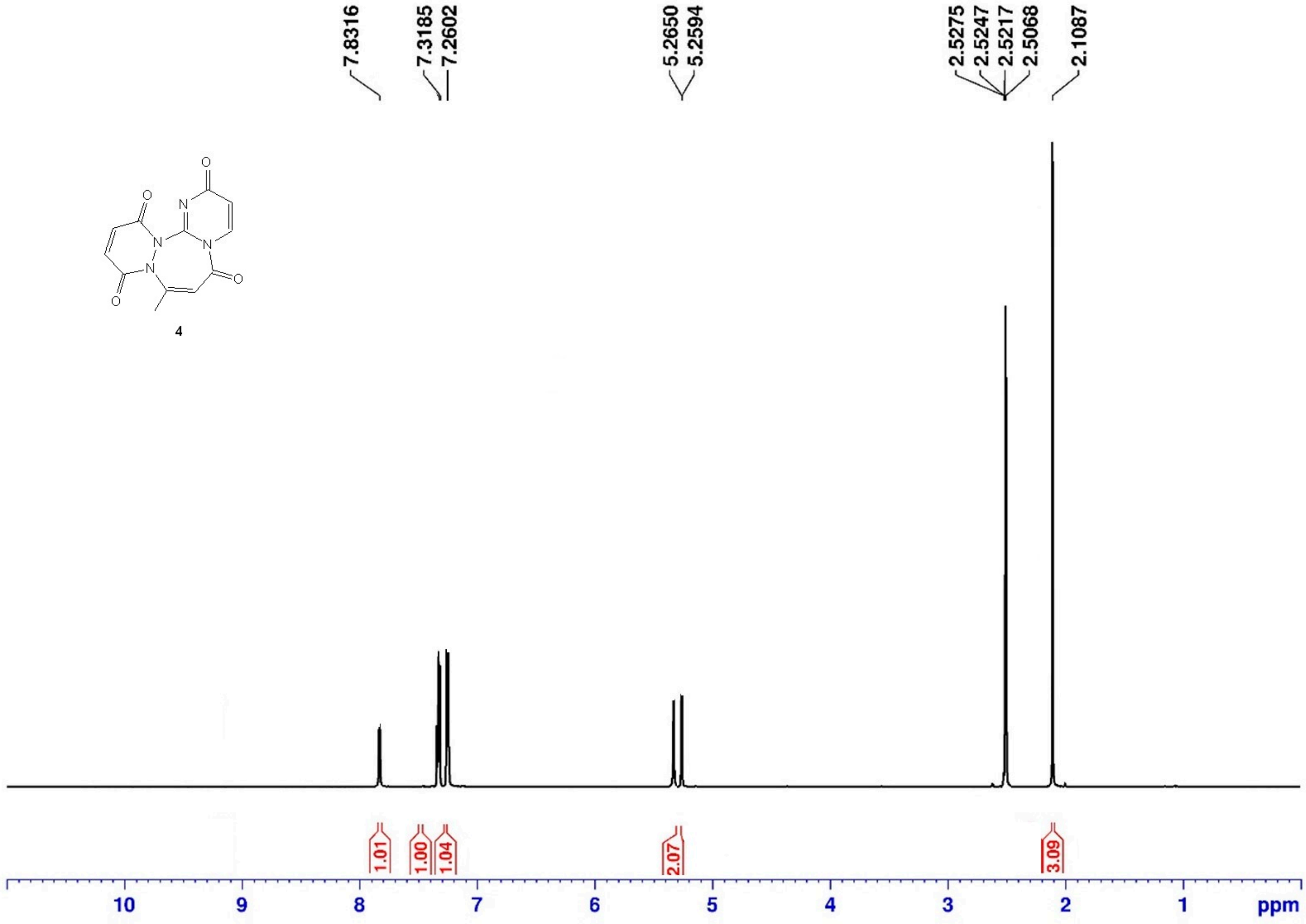


**Figure S3** $^{1}$H NMR Spectrum (500 MHz, DMSO-*d6*) of Compound **4**

***8-methyl-2H,6H-pyridazino[1,2-a] pyrimido[2,1-c] [1,2,4] triazepine-2,6,10,13-tetraone*** **(4)**

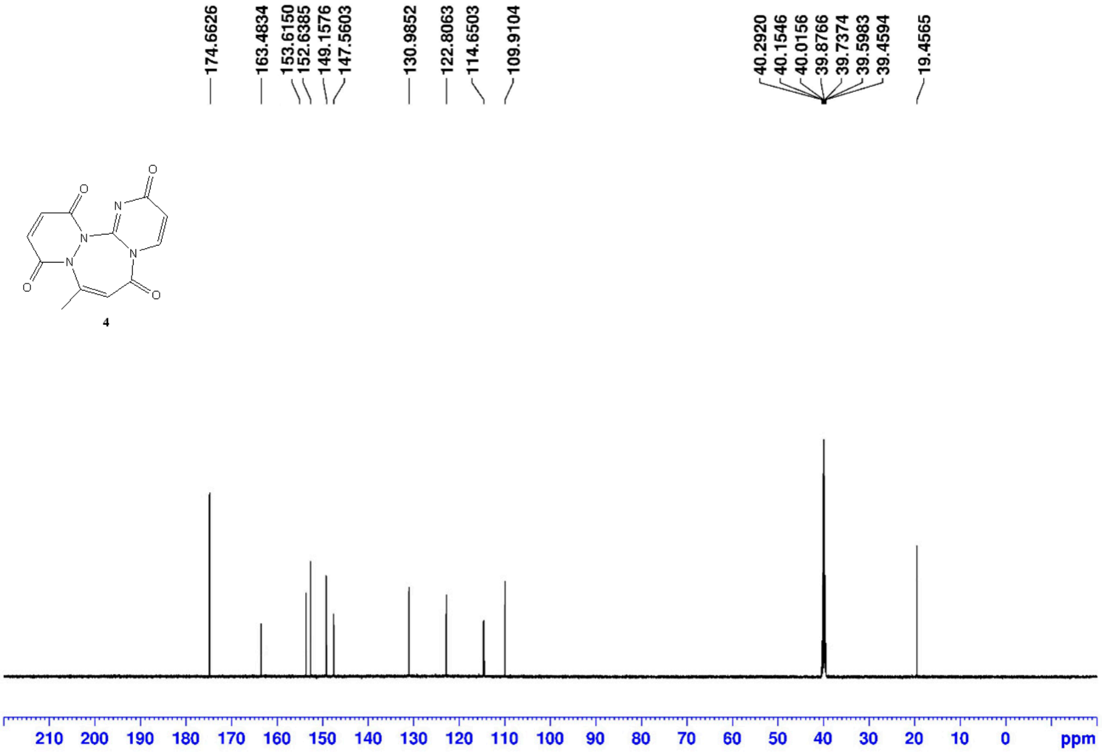


**Figure S4** $^{13}$C NMR Spectrum (125 MHz, DMSO-*d6*) of Compound **4**

***2H,6H-pyridazino[1,2-a] pyrimido[2,1-c] [1,2,4] triazepine-2,6,8,10,13(7H)-pentaone* (5)**

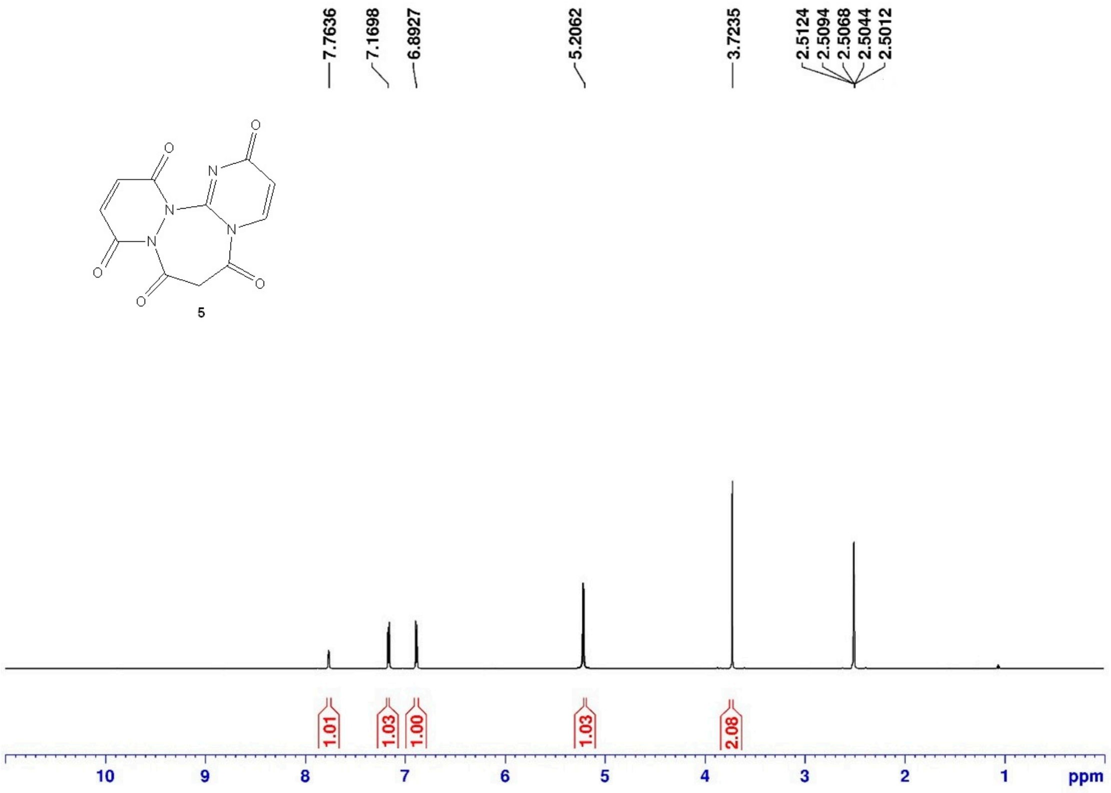


**Figure S5** $^{1}$H NMR Spectrum (500 MHz, DMSO-*d6*) of Compound **5**

*2H,6H-pyridazino[1,2-a] pyrimido[2,1-c] [1,2,4] triazepine-2,6,8,10,13(7H)-pentaone (5)*

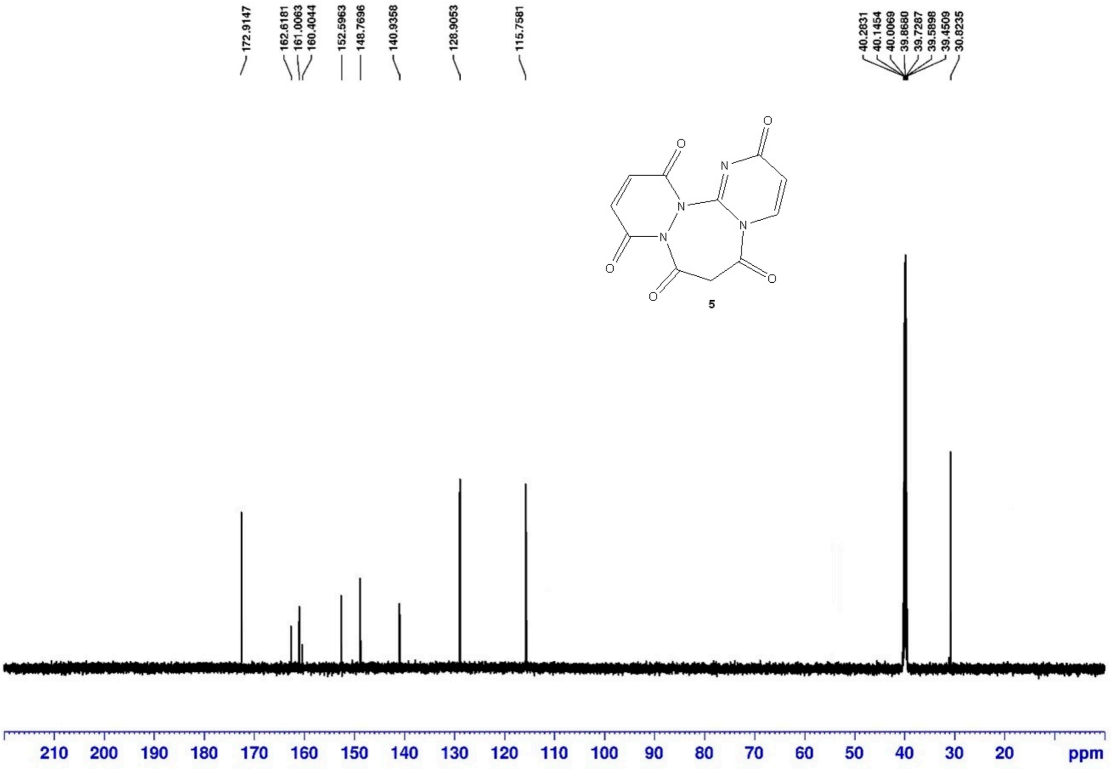


**Figure S6** $^{13}$C NMR Spectrum (125 MHz, DMSO-*d6*) of Compound **5**

***8-hydroxy-6,8-dimethyl-2H,8H-pyridazino[1,2-a] pyrimido[2,1-c] [1,2,4] triazepine-2,10,13-trione*** **(6)**

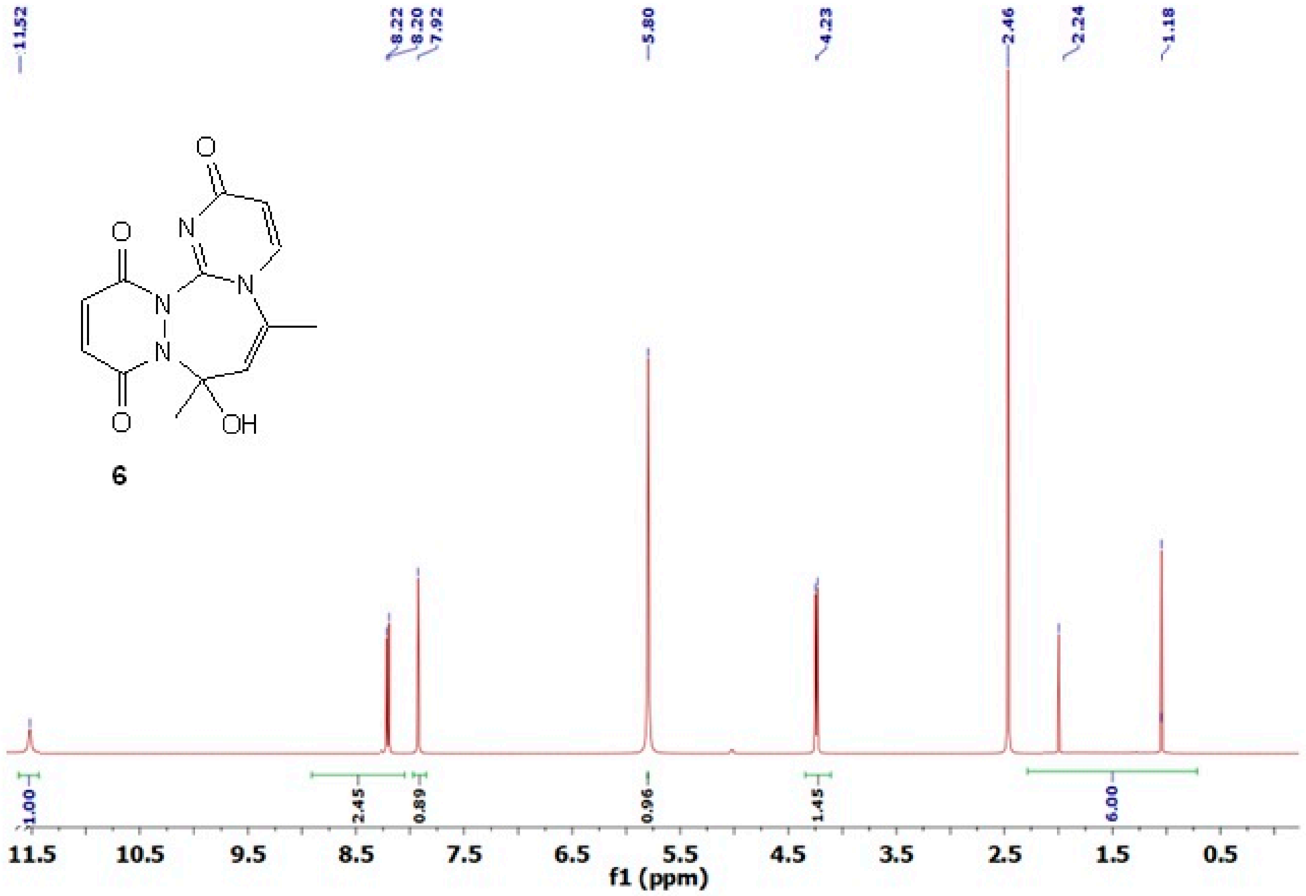


**Figure S7** $^{1}$H NMR Spectrum (500 MHz, DMSO-*d6*) of Compound **6**

*8-hydroxy-6,8-dimethyl-2H,8H-pyridazino[1,2-a] pyrimido[2,1-c] [1,2,4] triazepine-2,10,13-trione* (**6**)

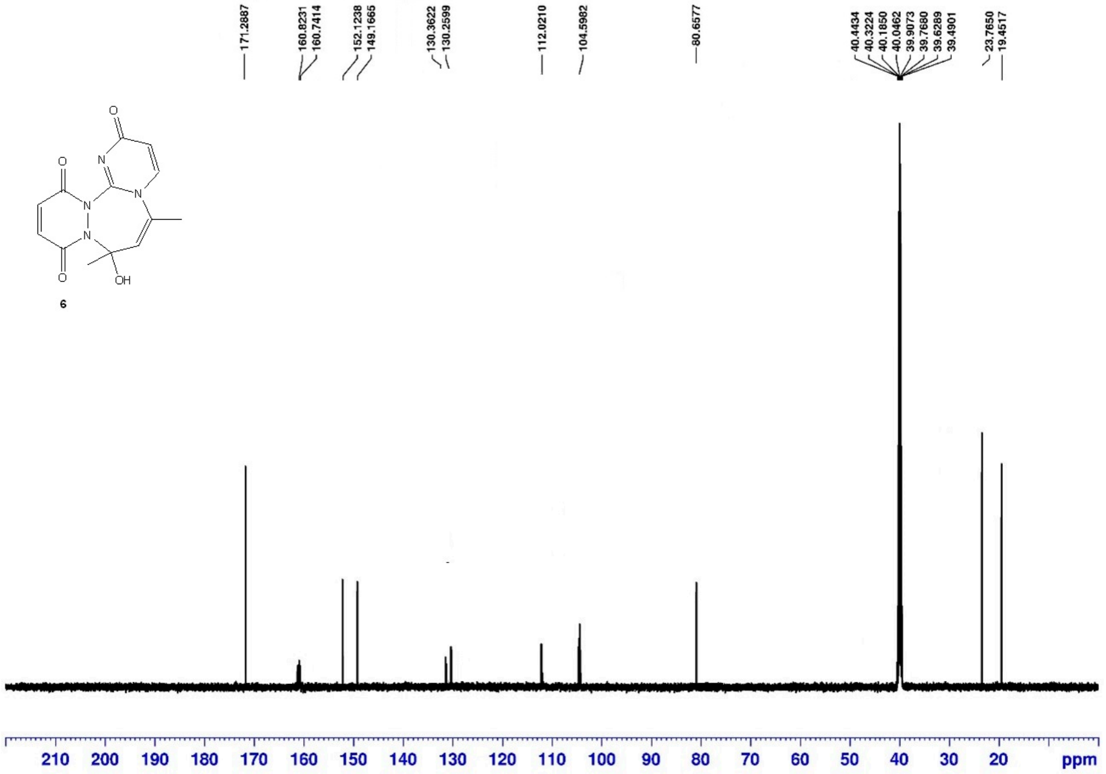


**Figure S8** $^{13}$C NMR Spectrum (125 MHz, DMSO-d6) of Compound **6**

***8-(chloromethylene)-6-methyl-2H,8H-pyridazino[1,2-a] pyrimido[2,1-c][1,2,4] triazepine-2, 10, 13-trione*** **(7)**

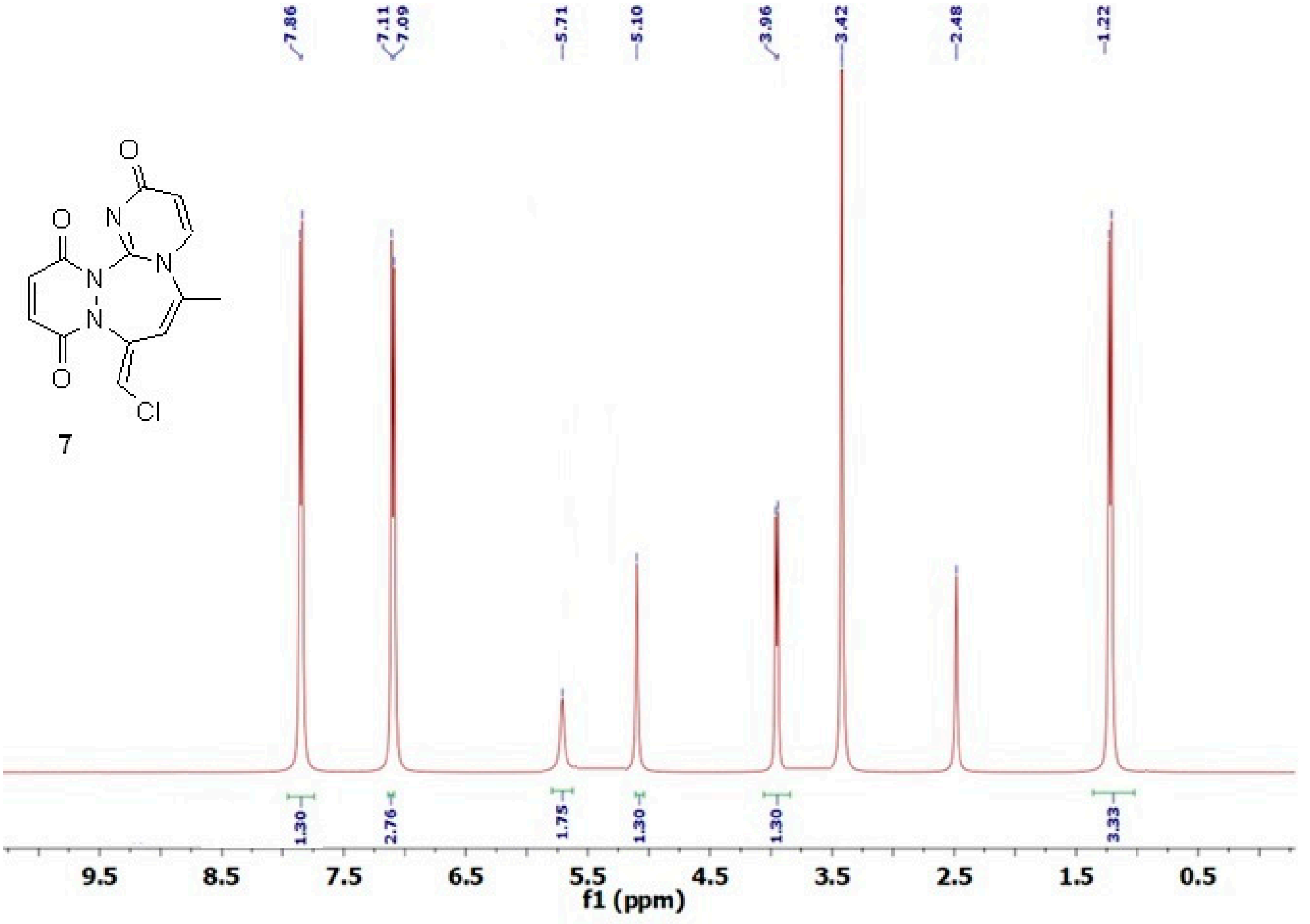


**Figure S9** $^{1}$H NMR Spectrum (500 MHz, DMSO-*d6*) of Compound **7**

***6,8-diethylidene-2H,6H,8H-pyridazino[1,2-c]pyrimido[2,1-e][1,3,4,6] oxatriazepine-2,10,13-trione*** **(8)**

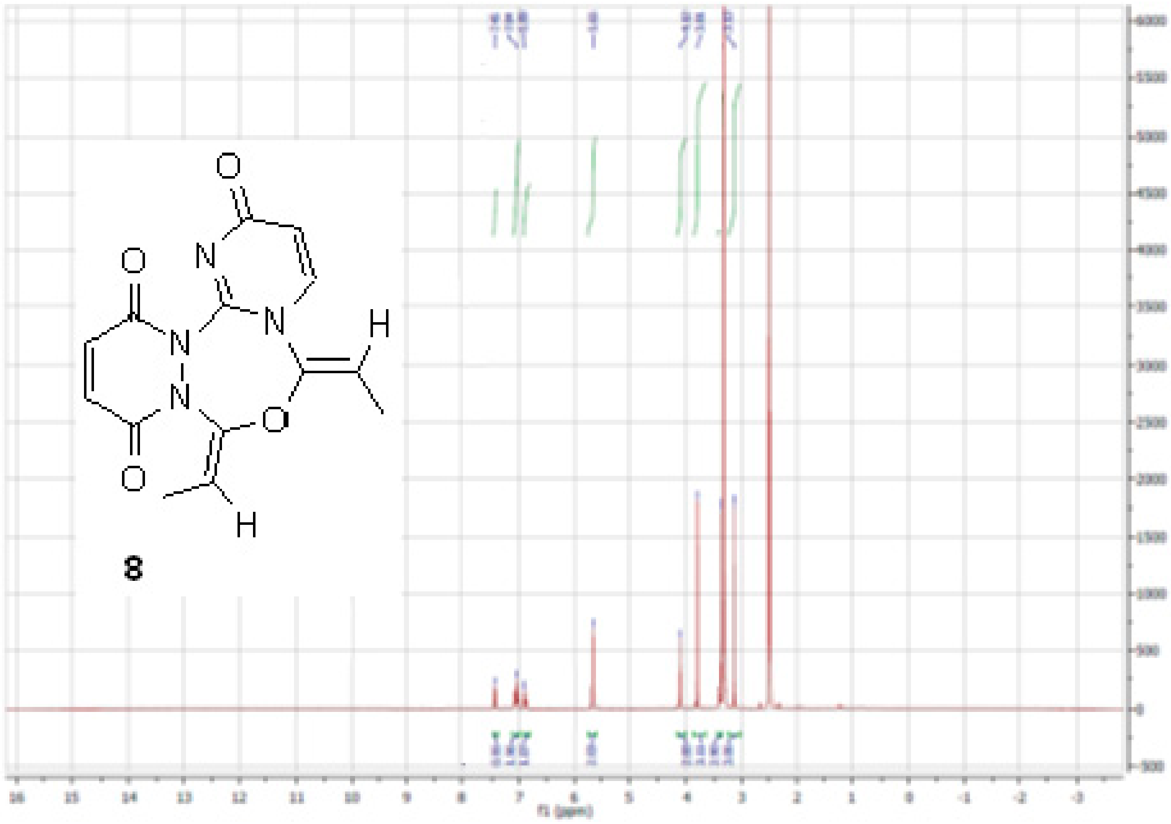

**Figure S10** $^1$H NMR Spectrum (500 MHz, DMSO-*d6*) of Compound **8**

***6,8-diethylidene-2H,6H,8H-pyridazino[1,2-c]pyrimido[2,1-e][1,3,4,6] oxatriazepine-2,10,13-trione* (8)**

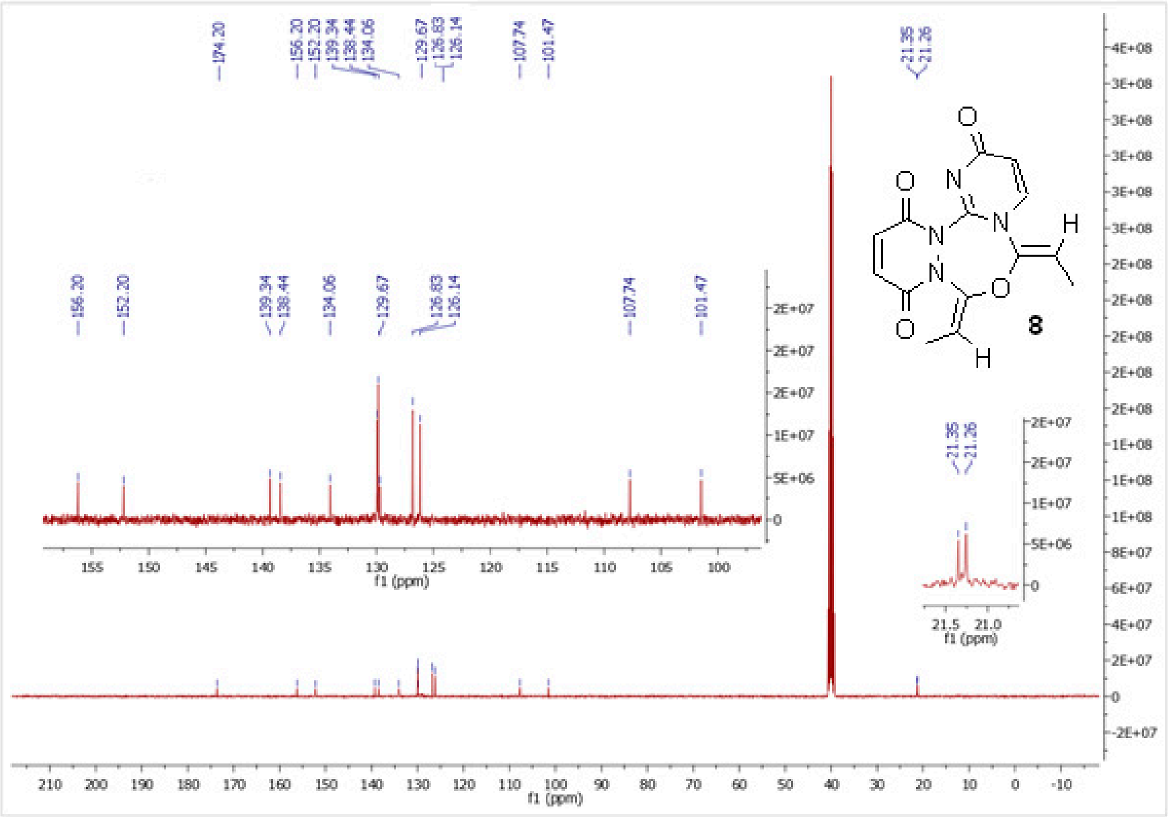

**Figure S11** $^{13}$C NMR Spectrum (125 MHz, DMSO-*d6*) of Compound **8**

***8-amino-2H,6H-pyridazino[1,2-a]pyrimido[2,1-c][1,2,4]triazepine-2,6,10,13-tetraone* (9)**

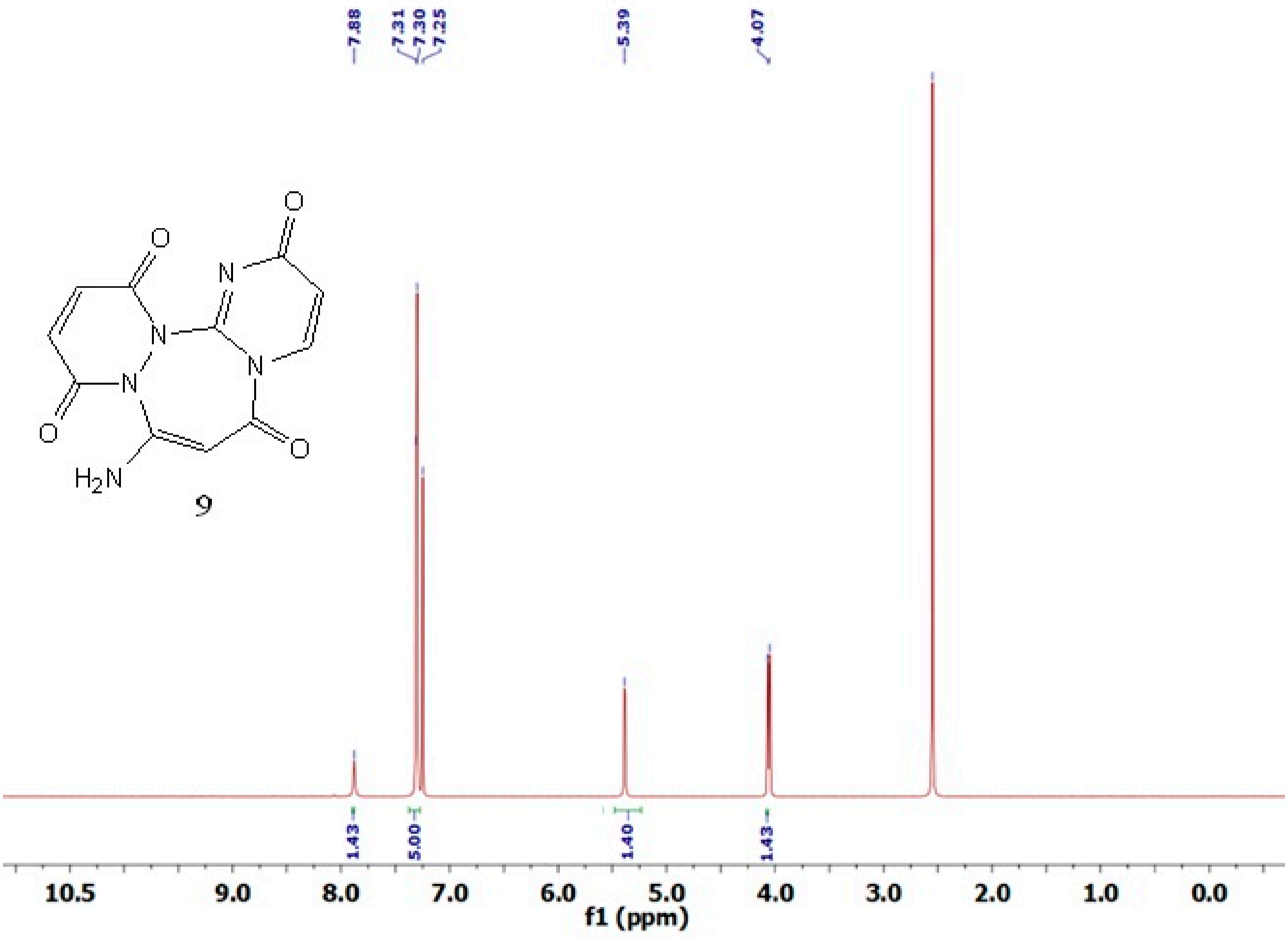


**Figure S12** $^{1}$H NMR Spectrum (500 MHz, DMSO-*d6*) of Compound **9**

***8-amino-2H,6H-pyridazino[1,2-a]pyrimido[2,1-c][1,2,4]triazepine-2,6,10,13-tetraone* (9)**

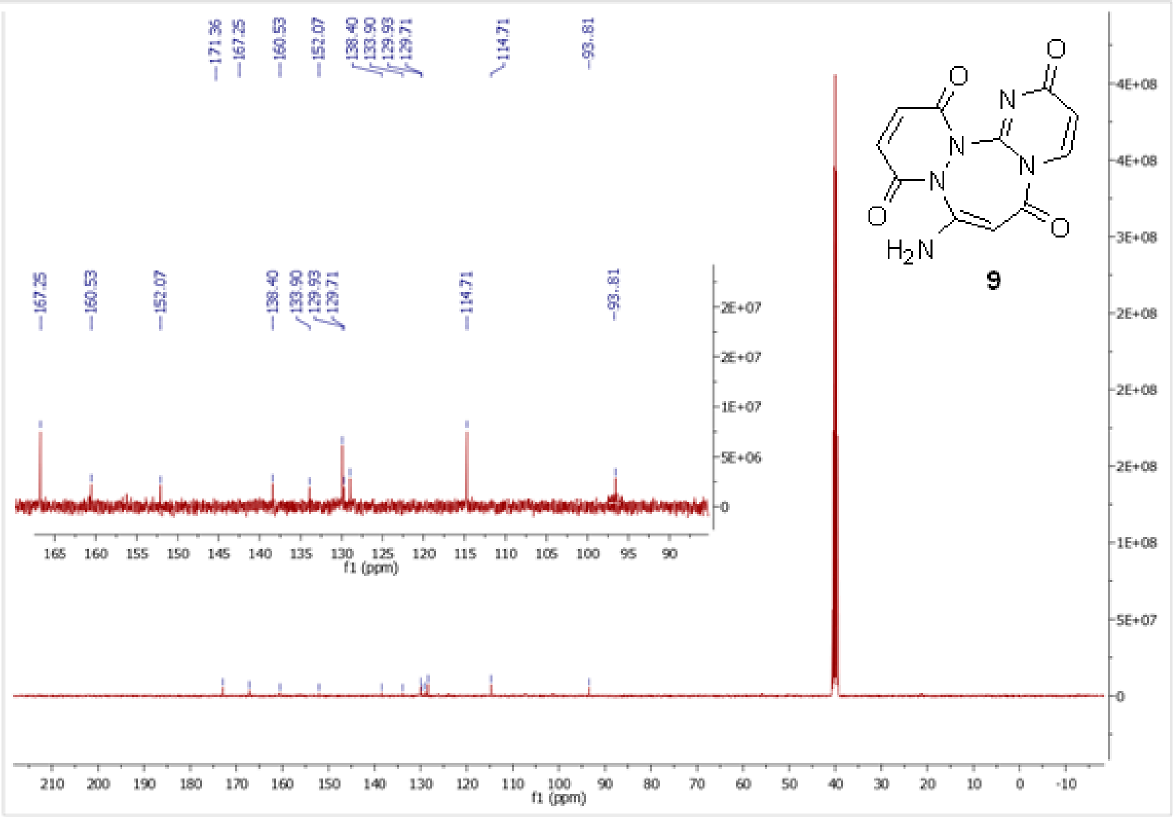


**Figure S13** $^{13}$C NMR Spectrum (125 MHz, DMSO-*d6*) of Compound **9**

***6-amino-8-imino-2H,8H-pyridazino[1,2-a]pyrimido[2,1-c][1,2,4]triazepine-2,10,13-trione* (10)**

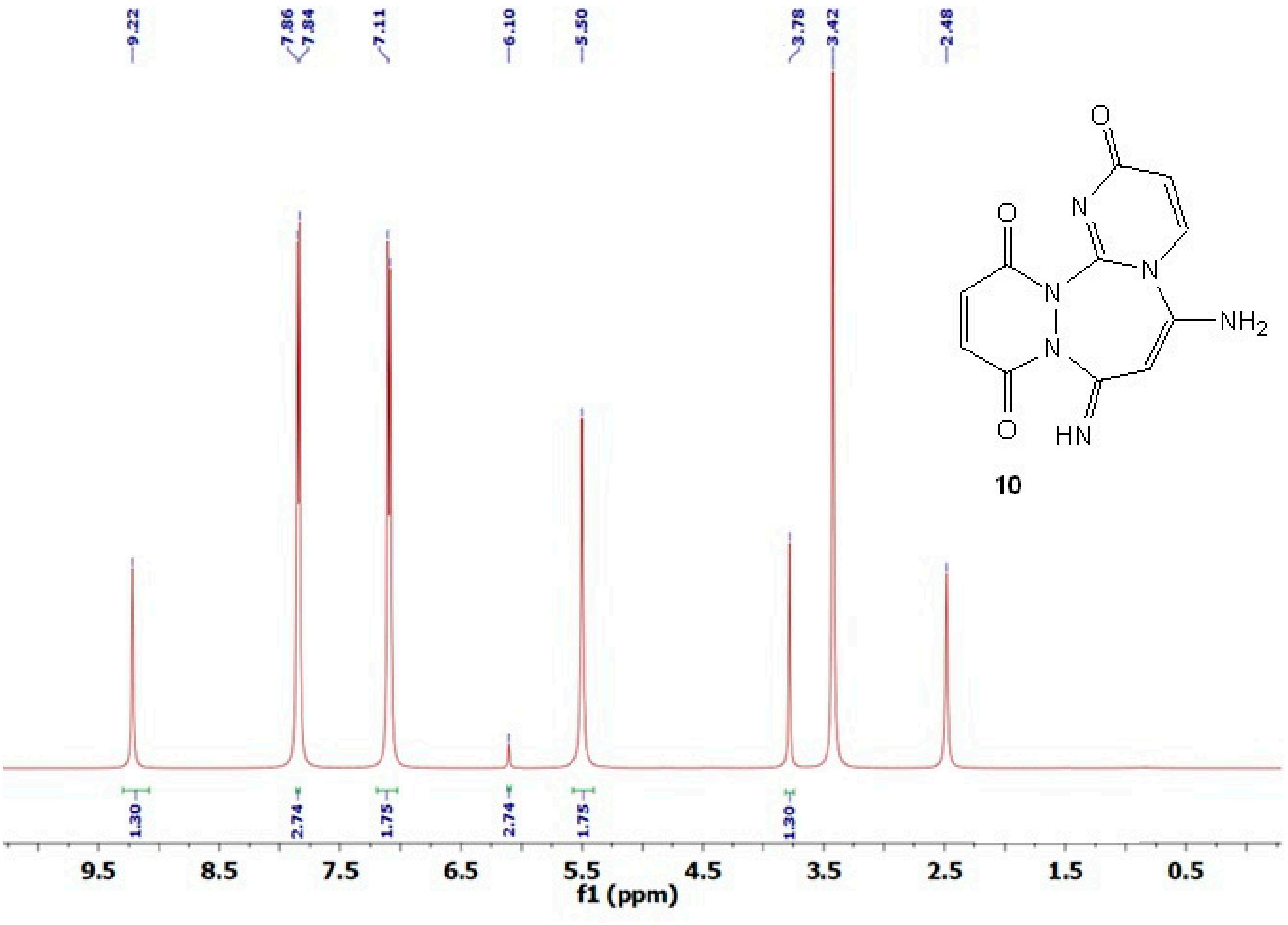


**Figure S14** $^1$H NMR Spectrum (500 MHz, DMSO-*d6*) of Compound **10**

***6-amino-8-imino-2H,8H-pyridazino[1,2-a]pyrimido[2,1-c][1,2,4]triazepine-2,10,13-trione*** **(10)**

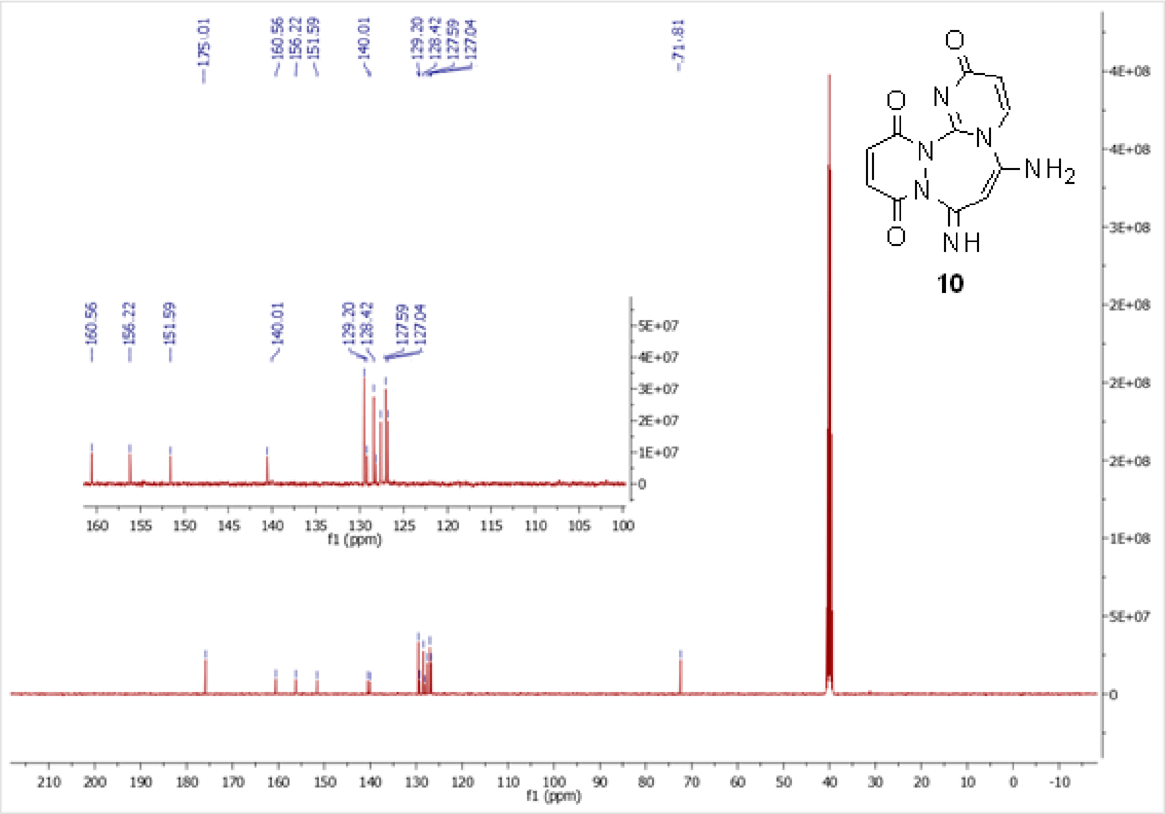

**Figure S15** $^{13}$C NMR Spectrum (125 MHz, DMSO-*d6*) of Compound **10**
